# A Targetron System for Gene Targeting in Thermophiles and Its Application in *Clostridium thermocellum*

**DOI:** 10.1371/journal.pone.0069032

**Published:** 2013-07-09

**Authors:** Georg Mohr, Wei Hong, Jie Zhang, Gu-zhen Cui, Yunfeng Yang, Qiu Cui, Ya-jun Liu, Alan M. Lambowitz

**Affiliations:** 1 Section of Molecular Genetics and Microbiology, Department of Chemistry and Biochemistry, Institute for Cellular and Molecular Biology, School of Biological Sciences, University of Texas at Austin, Austin, Texas, United States of America; 2 Shandong Provincial Key Laboratory of Energy Genetics, and Key Laboratory of Biofuels, Qingdao Institute of Bioenergy and Bioprocess Technology, Chinese Academy of Sciences, Qingdao, People’s Republic of China; 3 State Key Joint Laboratory of Environment, Simulation and Pollution Control, School of Environment, Tsinghua University, Beijing, People’s Republic of China; 4 University of Chinese Academy of Sciences, Chinese Academy of Sciences, Beijing, People’s Republic of China; University of Houston, United States of America

## Abstract

**Background:**

Targetrons are gene targeting vectors derived from mobile group II introns. They consist of an autocatalytic intron RNA (a “ribozyme”) and an intron-encoded reverse transcriptase, which use their combined activities to achieve highly efficient site-specific DNA integration with readily programmable DNA target specificity.

**Methodology/Principal Findings:**

Here, we used a mobile group II intron from the thermophilic cyanobacterium *Thermosynechococcus elongatus* to construct a thermotargetron for gene targeting in thermophiles. After determining its DNA targeting rules by intron mobility assays in *Escherichia coli* at elevated temperatures, we used this thermotargetron in *Clostridium thermocellum*, a thermophile employed in biofuels production, to disrupt six different chromosomal genes (*cipA*, *hfat*, *hyd*, *ldh*, *pta*, and *pyrF*). High integration efficiencies (67–100% without selection) were achieved, enabling detection of disruptants by colony PCR screening of a small number of transformants. Because the thermotargetron functions at high temperatures that promote DNA melting, it can recognize DNA target sequences almost entirely by base pairing of the intron RNA with less contribution from the intron-encoded protein than for mesophilic targetrons. This feature increases the number of potential targetron-insertion sites, while only moderately decreasing DNA target specificity. Phenotypic analysis showed that thermotargetron disruption of the genes encoding lactate dehydrogenase (*ldh*; Clo1313_1160) and phosphotransacetylase (*pta*; Clo1313_1185) increased ethanol production in *C. thermocellum* by decreasing carbon flux toward lactate and acetate.

**Conclusions/Significance:**

Thermotargetron provides a new, rapid method for gene targeting and genetic engineering of *C. thermocellum*, an industrially important microbe, and should be readily adaptable for gene targeting in other thermophiles.

## Introduction

Renewable fuels like bioethanol are urgently needed due to ever increasing global energy demands, limited quantities of fossil fuels, and climate change [1], [2]. Thermophiles with optimal growth temperatures of ∼60°C have been proposed as promising producers of low-cost bioethanol [1]–[3] because thermophilic microorganisms: (i) generally have low cellular growth yield and contain very stable enzyme systems [4]; (ii) usually degrade plant biomass and ferment many kinds of mono- or oligosaccharides [5], [6]; and (iii) grow at high temperatures, which reduce the risk of contamination and facilitate the removal of volatile end products, such as ethanol [3], [4]. *Clostridium thermocellum* is a thermophilic anaerobic bacterium well known for its robust cellulose-degrading system [7]. Hence, it is considered one of the most promising candidates for consolidated bioprocessing (CBP) of cellulolytic ethanol [8]. However, natural deficiencies have impeded industrial applications of *C. thermocellum* and other thermophiles. For instance, *C. thermocellum* is unable to utilize pentose, which is the main product of hemicelluloses, and its tolerance for ethanol and other hydrolysates is generally low [6], [8]. The recently determined genome sequences of *C. thermocellum* strains enable metabolic engineering by targeting specific genes and pathways to improve ethanol production. Although a gene disruption method based on homologous recombination has been developed for *C. thermocellum*, it is not widely used due to its requirements for high transformation frequencies and low gene disruption efficiency [9]–[12]. Thus, novel gene targeting methods are required for the efficient metabolic engineering of *C. thermocellum*, as well as for other industrially important thermophiles.

Targetrons are gene targeting vectors derived from mobile group II introns [13]–[15]. Their utility for gene targeting stems from their novel ribozyme-based DNA integration mechanism, termed “retrohoming”, which is mediated by a ribonucleoprotein (RNP) complex that contains the excised intron lariat RNA and an intron-encoded protein (IEP) with reverse transcriptase (RT) activity [16]. After being formed during RNA splicing, group II intron RNPs recognize DNA target sequences for intron insertion by using both the IEP and base pairing of the intron RNA [17]. For mesophilic group II introns, the IEP recognizes a small number of nucleotide bases (typically 4 to 6) in double-stranded DNA and helps promote DNA melting, enabling the intron RNA to base pair to the adjacent 11–14 nt region of the DNA strand encompassing the intron-insertion site [13], [15], [18], [19]. The intron RNA then uses its ribozyme activity to insert by reverse splicing directly into the DNA strand to which it is base paired, while the IEP cuts the opposite strand and uses the cleaved 3′ end as a primer for reverse transcription of the inserted intron RNA. The resulting intron cDNA is integrated into the genome by host enzymes [20]–[22]. Because the DNA target sequence is recognized largely by base pairing of the intron RNA, group II introns can be retargeted to insert into desired sites, simply by modifying the base-pairing sequences in the intron RNA. Gene targeting using mesophilic group II introns is highly efficient and specific, with targeting frequencies typically ranging from 1–100% without selection.

A targetron based on the *Lactococcus lactis* Ll.LtrB intron, which belongs to structural subclass IIA, has been widely used for gene targeting in different bacteria [14], [15], [23], [24], and recently, two other mobile group II introns, *Escherichia coli* EcI5 and *Sinorhizobium meliloti* RmInt1, which belong to a different intron subclass (IIB), were similarly adapted for gene targeting [25], [26]. In all three cases, targeted group II intron RNPs are expressed from a donor plasmid that is introduced into the bacteria by electroporation or conjugation [24]. Targetron donor plasmids typically use an inducible or constitutive promoter to express a precursor RNA containing the ribozyme portion of the intron (deleted for the intron ORF; denote I-ΔORF) flanked by 5′ and 3′ exons (E1 and E2, respectively), with the IEP expressed separately in tandem [13], [14], [26]. The I-ΔORF RNA splices more efficiently than does the full-length intron RNA, is resistant to degradation by cellular nucleases, and integrates stably into the genome, since it cannot be spliced or re-mobilized in the absence of the IEP. The intron can be targeted to insert in either the antisense or sense orientation relative to target gene transcription by selecting target sequences in opposite DNA strands. Targetrons that insert in the antisense orientation cannot be spliced and yield unconditional disruptions, whereas targetrons that insert in the sense orientation can be used to obtain conditional disruptions by linking their splicing to the expression of the IEP from a separate construct [23], [27]. Targeting frequencies in bacteria are generally high enough to detect desired integrations by colony PCR screening without selection [15], but genetic markers, including retrotransposition-activated markers (RAMs), can be inserted into the intron to select for desired integrations [28], [29]. Because mismatches between the intron RNA and DNA target site affect the *k*_cat_ as well as the *K*_m_ for the DNA integration reaction [30], group II intron insertion is highly specific, with Southern hybridizations generally showing just a single integration at the desired site [15].

The Ll.LtrB targetron has a broad host range and has been used for gene targeting in a variety of Gram-negative and Gram-positive bacteria, including *E. coli, Salmonella typhimurium, Shigella flexneri* [14]; *Lactococcus lactis* [27]; *Clostridium* spp. [29], [31]; *Staphylococcus aureus* [23], [32]; *Pseudomonas* spp. and *Agrobacterium tumefaciens* [24], [33]; *Azospirillum brasiliense* [34]; *Francisella tularensis* [35]; *Listeria monocytogene*s [36]; *Paenibacillus alvei* [37]; *Pasteurella multocida* [38]; *Ralstonia eutropha* [39]; *Staphylococcus saprophyticus* [40]; *Yersinia pseudotuberculosis* [41], [42]; *Sodalis glossinidius* [43]; and *Bacillus anthracis* [44]. A number of these bacteria had previously been intractable to gene targeting by other methods. Published applications of targetrons include site-specifically inserting a phage-resistance gene cloned within the intron at a regulatable chromosomal location in *L. lactis* [27]; inserting antigens and inactivating toxin genes in vaccine strains [31]; generating bacterial strains containing multiple insertions for high-level protein expression [45]; the identification of virulence factors and drug targets in pathogenic bacteria [46], [47]; and increasing the level of production of chemicals and biofuels, such as isobutanol and ethanol [48]–[50]. The ability to obtain multiple insertions, disruptions, and conditional disruptions at high frequency without selection is advantageous for synthetic and systems biology approaches for bacterial genetic engineering.

Group II introns that might be used to construct a thermotargetron have been identified in the genomes of a number of thermophiles [51]–[54]. Among them, the thermophilic cyanobacterium *Thermosynechococcus elongatus* contains 28 group IIB introns, which are closely related to each other and are thought to have evolved from a single ancestral intron that colonized this bacterium [51], [55]. Recently, we characterized the *T. elongatus* group II introns by retrohoming assays in *E. coli* at elevated temperatures and identified several introns that are actively mobile and thermophilic with retrohoming efficiencies of near 100% in plasmid-based assays at 48°C [55]. Here we developed one of these *T. elongatus* group II introns into the first thermotargetron and show that it can be used for efficient chromosomal gene targeting in *C. thermocellum* at high temperatures. Further, thermotargetron recognizes DNA target sites almost entirely by base pairing of the intron RNA with minimal recognition by the IEP, whose contribution to DNA melting appears to be largely dispensable at higher temperatures. This feature is advantageous for targeting short ORFs and small non-coding RNAs, but decreases target specificity, thus requiring greater attention to targetron design to avoid integration into closely matching off-target sites.

## Results

### Construction of the TeI3c/4c Thermotargetron

**Figure 1:**
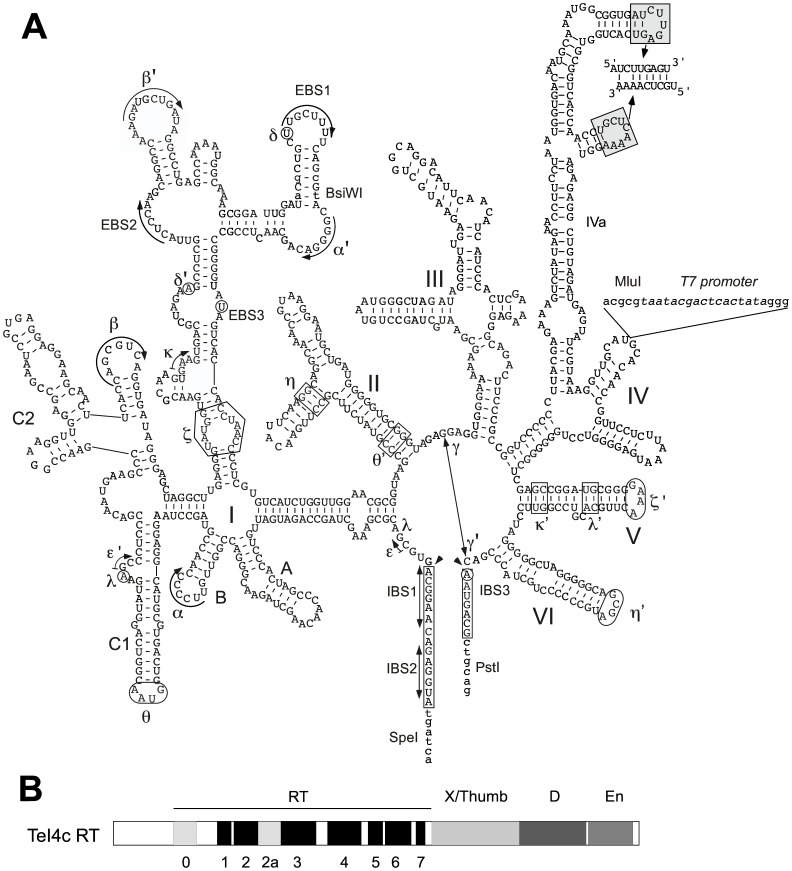
The *T. elongatus* TeI3c group II intron RNA and TeI4c RT components of the thermotargetron. (A) A secondary structure model of group II intron TeI3c showing modifications used for retrohoming assays and the construction of the thermotargetron. Nucleotide residues that differ from wild-type TeI3c are shown in lower case letters, exon sequences are boxed, and restriction sites used in plasmid constructions are in bold. The T7 promoter sequence inserted in intron domain IV for plasmid-based retrohoming assays in *E. coli* (Fig. 2) is in italics. Greek letters denote sequence elements involved in predicted tertiary structure interactions [16]. The loops of two stem-loop structures in subdomain DIVa (shaded boxes) can potentially base pair to form the pseudoknot shown. (B) Schematic representation of the TeI4c RT, which splices and mobilizes group II intron TeI3c. Conserved protein domains are: RT, containing conserved amino acid sequence blocks RT1–7 characteristic of the finger and palm regions of retroviral and other RTs; X/Thumb; D, DNA binding; and En, DNA endonuclease. RT-0 and -2a (hatched) are additional conserved sequence blocks found in the RT domains of non-LTR-retroelement RTs [16], [72], [73]. The RT and X/Thumb domains function together in reverse transcription and specific binding of the intron RNA, which stabilizes the catalytically active RNA structure for RNA splicing and reverse splicing of the intron into the DNA target site; domain D contributes to DNA target site recognition; and the En domain cleaves the opposite strand of the DNA target site to generate the primer for reverse transcription of the reverse-spliced intron RNA.

To construct a thermotargetron, we focused initially on the *T. elongatus* group II intron TeI4h*, a derivative of TeI4h in which we had engineered modifications of both the intron RNA and RT that together increased its retrohoming efficiency to near 100% in an *E. coli* plasmid assay at 48°C [55]. We found, however, that TeI4h* is not easily retargetable, likely due to difficulties with its exon-binding site 2 (EBS2), one of the sequence elements that base pairs to the DNA target site. Unlike in other group II introns, the TeI4h EBS2 base pairs unpredictably to DNA target sites in different registers, possibly a mechanism that enables this intron to proliferate by inserting into a larger number of DNA sites in its host genome (unpublished data). We then switched to another *T. elongatus* intron TeI3c (Fig. 1A). TeI3c is a naturally ORFless group II intron that inserted into the RT ORF of another mobile group II intron (denoted TeI4c), a configuration known as a “twintron”. We found that the TeI4c RT (Fig. 1B) could support independent retrohoming of both group II introns comprising the twintron and surprisingly, mobilized the secondary ORFless intron TeI3c more efficiently than the primary intron TeI4c in which it is encoded [55].

**Figure 2:**
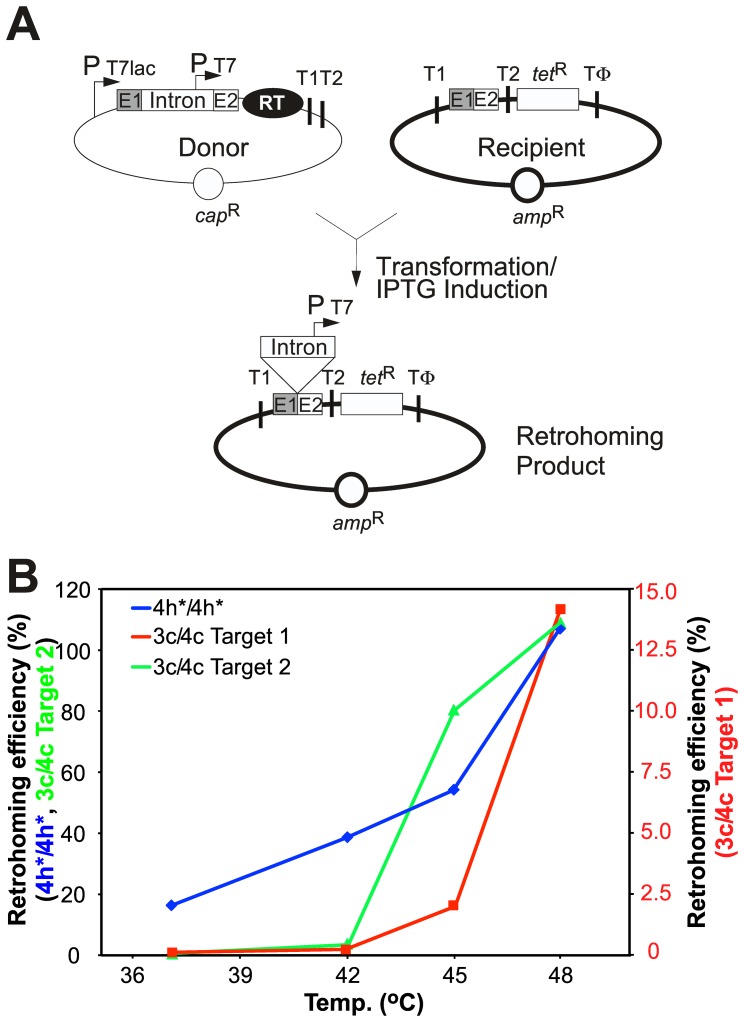
Temperature profiles of retrohoming by thermophilic group II introns in *E. coli*. (A) *E. coli* plasmid-based retrohoming assay [13], [14], [55]. The Cap^R^ intron-donor plasmid uses a T7lac promoter (P_T7lac_) to express a group II intron RNA with short flanking 5′ and 3′ exons (E1 and E2, respectively) and the group II RT cloned downstream of E2. The group II intron, which has a T7 promoter sequence (P_T7_) inserted near its 3′ end, integrates into a target site (the ligated E1–E2 sequence) cloned in a compatible Amp^R^ recipient plasmid upstream of a promoterless *tet* ^R^ gene, thereby introducing the T7 promoter and activating that gene. The assays are done in *E. coli* HMS174(DE3), which contains an IPTG-inducible T7 RNA polymerase. Intron expression is induced with IPTG, and mobility efficiencies are calculated as the ratio of (Tet^R^+Amp^R^)/Amp^R^ colonies. (B) Temperature dependence of intron retrohoming. Retrohoming assays were done as described in panel (A) in *E. coli* HMS174(DE3), using intron-donor plasmids pACD2X-TeI4h*/4h*, pACD2X-TeI3c/4c, and a derivative of pACD2X-TeI3c/4c that has been retargeted to insert into a site in the *E. coli lacZ* gene (see Fig. 4). Targetron expression was induced with 500 µM IPTG for 1 h at different temperatures. Recipient plasmids contain the DNA target sites for each intron from positions −30 to +15 from the intron-insertion site. Target sites 1 and 2 for TeI3/4c are the native target site for the wild-type intron and the *lacZ* site target site for the retargeted intron, respectively. The figure shows data from a single experiment, which was repeated with similar results.

We evaluated the performance of potential thermotargetron constructs by using a previously developed *E. coli* plasmid assay in which a group II intron with a phage T7 promoter sequence inserted near its 3′ end is expressed from a donor plasmid and retrohomes into a target site cloned in a recipient plasmid upstream of a promoterless *tet* ^R^ gene, thereby activating that gene (Fig. 2A). For thermotargetrons, the assays were done at elevated temperature in *E. coli* HMS174(DE3), which is RecA^−^ and encodes an isopropyl β-D-1 thiogalactopyranoside (IPTG)-inducible T7 RNA polymerase. The Cap^R^ intron-donor plasmid uses a T7lac promoter (P_T7lac_) to express the group II intron RNA and flanking 5′ and 3′ exons (E1 and E2, respectively) with a T7 promoter sequence (P_T7_) inserted in domain IV of the intron RNA, and the intron-encoded RT expressed separately from downstream of E2, the same configuration used for mesophilic targetrons [13], [14]. The Amp^R^ recipient plasmid contains the intron target site (the ligated E1–E2 sequence from positions −30 to +15 from the intron-insertion site) cloned upstream of the promoterless *tet* ^R^ gene. After insertion of the intron containing the T7 promoter sequence into the DNA target site, bacteria in which retrohoming occurred are readily selected by tetracycline-resistance, and mobility efficiencies are quantified as the ratio of (Tet^R^+Amp^R^)/Amp^R^ colonies.

We used this plasmid assay to compare the retrohoming efficiencies of the targetron constructs TeI4h*/4h* (TeI4h*-ΔORF intron RNA and TeI4h* RT) and TeI3c/4c (TeI3c RNA and TeI4c RT) at different induction temperatures (Fig. 2B). Both the native TeI3c intron with its native target site (target 1) and a retargeted TeI3c intron that inserts into a different target site (target 2) with higher retrohoming efficiency were tested. Unlike the mesophilic Ll.LtrB group II intron, whose retrohoming efficiency decreases at temperatures above 37°C [55], the retrohoming efficiencies of both the TeI4h*/4h* and TeI3c/4c targetrons increased at higher temperatures. Notably, while the retrohoming efficiency of the TeI4h*/4h* targetron increased progressively from ∼20% at 37°C to near 100% at 48°C, the native and retargeted TeI3c/4c targetrons showed virtually no retrohoming at 37°C, but a sharp increase in retrohoming efficiency at temperatures >42°C up to 100% for the retargeted TeI3c intron at 48°C.

### Determination of DNA Targeting Rules and Construction of Thermotargetron Expression Plasmids

The ability to target group II introns for efficient insertion into different target sites is based upon their use of both the IEP and base pairing of the intron RNA to recognize DNA target sequences, with the base-pairing interactions between the intron RNA and DNA target site providing most of the DNA target specificity [13], [15], . In the case of the mesophilic group II introns Ll.LtrB and EcI5, the IEP critically recognizes three to five nucleotide bases in the distal 5′-exon region of the DNA target site upstream of IBS2 and a smaller number of nucleotide bases in the 3′ exon [16]. For Ll.LtrB, IEP recognition of the distal 5′-exon region has been shown to promote local DNA melting, enabling the intron RNA to base pair to the adjacent DNA target sequence, while IEP recognition of the 3′ exon is required specifically for IEP cleavage of the bottom strand to generate the primer for target DNA-primed reverse transcription of the reverse spliced intron [18], [19].

**Figure 3:**
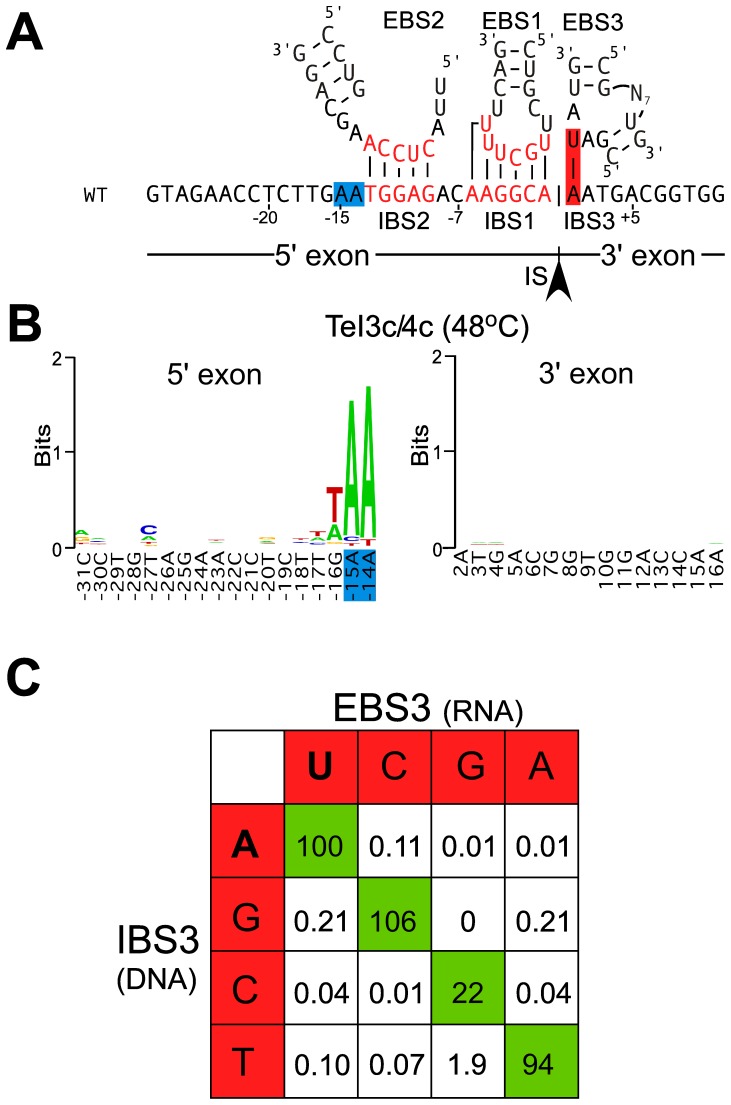
DNA target site recognition by thermotargetron TeI3c/4c. (A) DNA target site for group II intron TeI3c showing positions recognized by the IEP (blue) and intron RNA base pairing (red). IBS1, 2, and 3 denote intron-binding sites 1, 2, and 3 in the DNA target site, and EBS1, 2, and 3 denote exon-binding sites 1, 2, and 3 located in three different regions of the intron RNA. The arrowhead indicates the intron-insertion site (IS). (B) Target site positions recognized by the TeI4c RT. Nucleotide residues recognized by the TeI4c RT were identified in a selection experiment in *E. coli* HMS174(DE3) with IPTG induction at 48°C for 1 h using the donor plasmid pADC2X-TeI3c/4c and a recipient plasmid library with randomized nucleotide residues at positions −35 to −13 and +2 to +20. After plating on LB medium containing antibiotics, Amp^R^+Tet^R^ colonies were analyzed by colony PCR and sequencing of the 5′- and 3′-integration junctions to identify nucleotide residues in active target sites. The WebLogo representation [74] depicts nucleotide frequencies at each randomized position in 105 selected target sites, corrected for biases in the initial pool based on sequences of 100 randomly chosen recipient plasmids. The x-axis shows the sequence of the intron-insertion site in the *T. elongatus* genome, with blue residues highlighting the positions recognized by the IEP. The Figure was redrawn from [55]. (C) Retrohoming efficiency of the TeI3c/4c targetron with different EBS3/IBS3 pairings between the intron RNA and DNA target site. Retrohoming assays were done in *E. coli* HMS174(DE3) with IPTG induction for 1 h at 48°C with all possible combinations of donor plasmids pACD-TT1A, pACD-TT1C, pACD-TT1G, or pACD-TT1T [EBS3(RNA)] and recipient plasmids pBRR-3c (WT, IBS3A), pBRR-3cC, pBRR-3cG, or pBRR-3cT [IBS3(DNA)]. The grid shows mobility efficiencies for each combination of nucleotides at the EBS3 position in the intron RNA and the IBS3 position in the DNA target site. The wild-type U-A pairing is indicated in bold letters. The data are from a single experiment, which was repeated with similar results.

A model for DNA target site recognition by TeI3c/4c RNPs is shown in Figure 3A. To identify critical bases recognized by the IEP component of TeI3c/4c RNPs, we previously carried out an *in vivo* selection experiment using the same *E. coli* plasmid-based retrohoming assay at 48°C, but with a recipient plasmid that contains randomized sequences in the regions recognized by the IEP upstream and downstream of the IBS sequences [55]. We then isolated a collection of Tet^R^ colonies in which the intron had inserted into the recipient plasmid and sequenced the randomized regions to determine nucleotide frequencies in active target sites. The data from these selections, displayed in WebLogo format in Figure 3B, showed that the IEP strongly recognizes only the two A residues at positions −14 and −15 upstream of IBS2. The selections also showed a preference for A/T-rich sequences upstream of the region recognized by base pairing, presumably reflecting that such A/T-rich sequences facilitate DNA melting for intron RNA base pairing to the DNA target site.

TeI3c contains three sequence elements characteristic of subgroup IIB introns that contribute to DNA target site recognition by base pairing with sequences in the 5′ and 3′ exons flanking the intron-insertion site [16]. These sequence elements are denoted exon-binding sites 1, 2, and 3 (EBS1, 2, and 3), and the complementary sequences in the DNA target site are denoted intron-binding sites 1, 2, and 3 (IBS1, IBS2, and IBS3; Fig. 3A). The same EBS1, EBS2, and EBS3 sequences in the intron RNA also base pair with IBS1, IBS2, and IBS3 sequences in the 5′ and 3′ exons of the precursor RNA to position the exons at the group II intron RNA active site for RNA splicing (Fig. 1A).

Mesophilic group II introns are retargeted with the aid of a computer algorithm that scans the target sequence for the best matches to nucleotide residues recognized by the IEP and then designs primers for modifying the EBS sequences in the intron RNA to base pair to the IBS sequences in the DNA target site [15]. The IBS sequences in the 5′ and 3′ exons of the donor plasmid must also be modified to be complementary to the retargeted EBS sequences for efficient RNA splicing. To facilitate the retargeting of TeI3c, we constructed donor plasmids that have a unique SpeI site in exon 1 upstream of IBS2 and a unique BsiWI site within the intron downstream of EBS1, enabling the swapping in of a short (∼0.4-kb) PCR product containing both the retargeted EBS1 and EBS2 sequences and complementary IBS1 and IBS2 sequences in the 5′ exon of donor plasmid, which are required for RNA splicing (see Materials and Methods).

The IBS3 residue in the 3′ exon of the donor plasmid, which must also be complementary to EBS3 residue in the precursor RNA for efficient RNA splicing, is too distant from the other sequences to change in the same PCR step. Thus, to enable targeting of DNA sites with different IBS3 residues, we constructed four different donor plasmids with four different EBS3 residues and complementary IBS3 residues. These plasmids are named pACD2-TT1A, C, G, and T according to the identity of the IBS3 residue that can be targeted in the DNA target site. An experiment in which we compared retrohoming efficiencies of these four donor plasmids with target sites containing different IBS3 residues in every possible combination demonstrated that the EBS3 RNA/IBS3 DNA pairing contributes substantially to retrohoming efficiency and provided quantitative information about the relative efficiencies of different Watson-Crick and wobble pairings at this position (Fig. 3C).

### Targeting of the *E. coli lacZ* Gene

**Figure 4:**
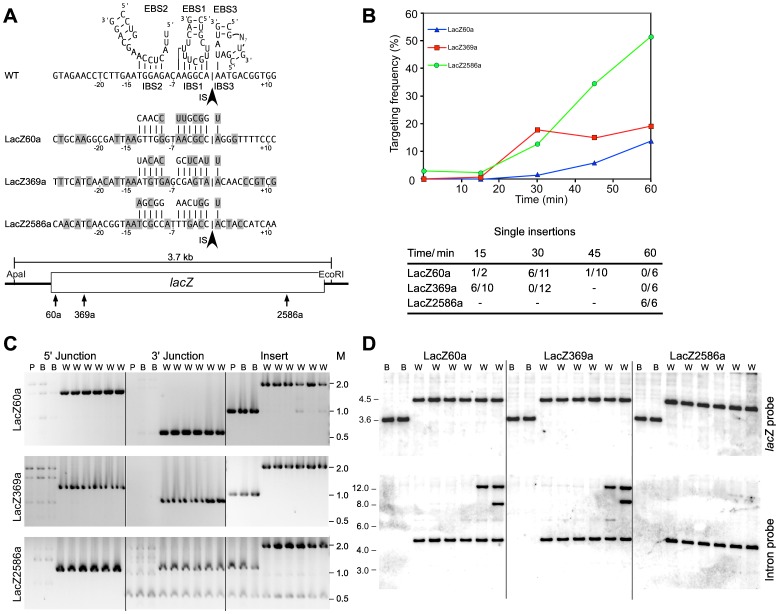
Targeted disruption of the *E. coli lacZ* gene at 48°C. (A) DNA target sequences and EBS/IBS interactions for thermotargetrons designed to insert into the *E. coli lacZ* gene. The wild-type target sequence and EBS/IBS interactions are shown above for comparison. The arrowhead indicates the intron-insertion site (IS), and gray shading highlights nucleotide residues in the *lacZ* target sites that match those in the wild-type target site. The schematic of the *lacZ* gene below shows the location of the targetron-insertion sites and the flanking ApaI and EcoRI sites used for Southern hybridizations. (B) Time course of *lacZ* targeting. After inducing thermotargetron expression in *E. coli* HMS174(DE3) with 500 µM IPTG at 48°C, *lacZ* targeting frequencies were determined by blue-white screening on LB+X-Gal agar plates. The Table shows the fraction of white colonies found by Southern hybridization to contain a single targetron insertion at the desired site. (C) PCR analysis. Eight colonies (two blue (B) and six white (W)) were picked for each targetron and compared to the parental *E. coli* HMS174(DE3) strain (P) in three PCRs with primers that flank the targetron-insertion site to detect the targetron insert or amplify the 5′- or 3′-integration junctions (Materials and Methods). (D) Southern hybridization analysis of two blue (B) and six white (W) colonies after induction of targetron expression for 15 or 30 min (LacZ60a and LacZ369a) or 1 h (LacZ2586a) at 48°C The blots show ApaI+EcoRI-digested chromosomal DNA hybridized with ^32^P-labeled probes for the TeI3c intron (nucleotides 1–342) or *lacZ* gene (nucleotides 30–1850). The *lacZ* probe hybridizes to a 3.7-kb band containing the wild-type *lacZ* gene in blue colonies and to a 4.5-kb band containing the *lacZ* gene with the inserted targetron in white colonies. The intron probe hybridizes to the same 4.5-kb band in the white colonies. Additional bands due to off-target integrations are observed in some white colonies.

We next tested whether the targeting rules determined above could be used to target the TeI3c intron to insert into sites within the *E. coli* chromosomal *lacZ* gene, whose disruption can be scored readily by blue-white screening. To identify potential targetron-insertion sites, we scanned the *lacZ* coding sequence using the simple search sequence WAA, where W is an A or T residue and the two A residues correspond to DNA target site positions −14 and −15 that are recognized by the IEP. Choosing from 160 such sites in the *lacZ* gene, we constructed targetrons LacZ60a, 369a and 2586a, which are directed to three sites in the antisense strand to give unconditional disruptions and have an A residue at IBS3 (Fig. 4A). (Targetrons are named according to the 5′-nucleotide residue of their insertion site in the *lacZ* ORF, with “a” or “s” indicating the antisense/bottom or sense/top strands). The targetrons were constructed in the intron-donor plasmid pACD-TT1A by replacing the wild-type SpeI-BsiWI fragment with a PCR-generated fragment that changed EBS1 positions −1 to −6 and EBS2 positions −9 to −13 to be complementary to the corresponding positions of the DNA target site (see Materials and Methods). The retargeted donor plasmids were transformed into *E. coli* HMS174(DE3) and induced with IPTG at 48°C for times ranging from 15 min to 1 h. The cells were then plated at different dilutions on X-gal plates, and targeting frequencies were quantified by blue-white screening.

For all three targetrons, the targeting frequencies measured by the percentage of white colonies increased with longer IPTG induction times from 0–2% at 15 min to 14–51% at 1 h (Fig. 4B). Colony PCR and sequencing of the PCR products confirmed that all tested white colonies contained the full-length targetron inserted precisely at the expected site in the *lacZ* gene, while all tested blue colonies lacked targetron insertions in *lacZ* (Fig. 4C). Southern blots of genomic DNA hybridized with a ^32^P-labeled intron probe identified disruptants with a single targetron insertion at the desired site for LacZ60a after a 15- or 30-min IPTG induction; for LacZ369a after a 15-min IPTG induction; and for LacZ2586a after a 60-min IPTG induction (Fig. 4D). However, while targetron LacZ2586a gave only single insertions at the expected site even after the longest induction time (1 h), an increasing proportion of the disruptants obtained with the LacZ60a and LacZ369a targetrons at longer induction times showed an additional 1 or 2 bands that hybridized with the intron probe, indicating off-target insertions. Such off-target insertions are rarely seen for the Ll.LtrB or EcI5 targetrons (*cf.*, [15], [25]) and likely reflect the smaller number of target site positions recognized by the TeI4c IEP (see above). Thus, additional precautions may be necessary to obtain desired single insertions with thermotargetrons (see Discussion).

### Targeting of *C. thermocellum* Chromosomal Genes

**Figure 5:**
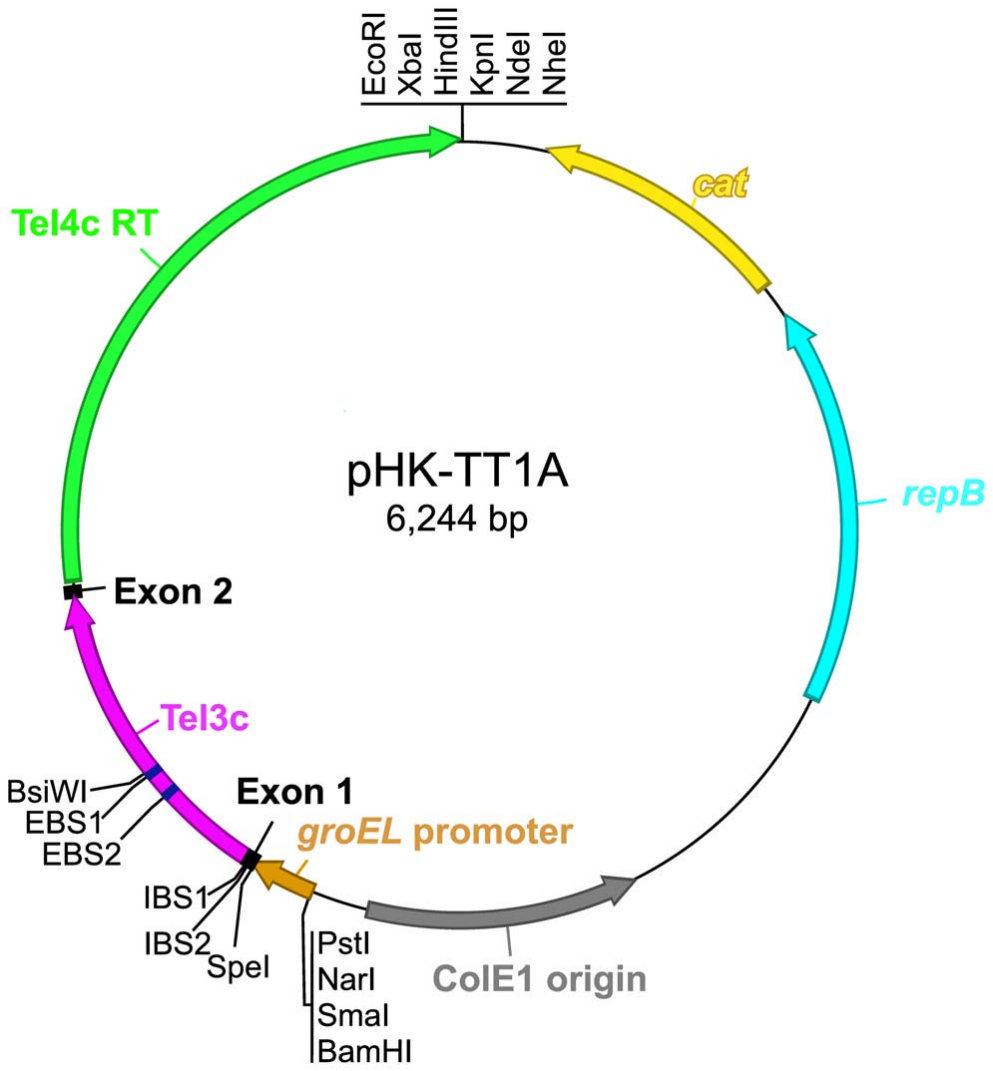
Map of plasmid pHK-TT1A used for thermotargtetron expression in *C. thermocellum*. The plasmid uses a *C. thermocellum groEL* promoter to express a thermotargetron cassette consisting of the *T. elongatus* TeI3c group II intron and flanking exon sequences followed by an ORF encoding the TeI4c RT. The targetron expression cassette is cloned in the *E. coli/C. thermocellum* shuttle vector pHK, which was derived from pNW33N (BGSC) and contains replication origins from *E. coli* plasmid pUC19 (ColE1) and *Geobacillus stearothermophilus* plasmid pTHT15 (RepB), as well as a chloramphenicol acetyltransferase (*cat*) gene from *Staphylococcus aureus* plasmid pC194 that has been used for selections in thermophiles at temperatures of 50–55°C [59], [60].

To test the function of the thermotargetron in a thermophile, we targeted chromosomal genes in *Clostridium thermocellum*, an organism that is used in biofuels production and has an optimal temperature range of 50–69°C [56], [57]. For these experiments, we constructed the thermotargetron donor plasmid pHK-TT1A in which the thermotargetron is expressed by using the constitutive promoter of the *C. thermocellum groEL* gene (Fig. 5) [58]. The thermotargetron expression cassette with the *groEL* promoter was cloned in an *E. coli/C. thermocellum* shuttle vector denoted pHK, a derivative of pNW33N (BGSC) containing replication origins from *Escherichia coli* plasmid pUC19 (ColE1) and *Geobacillus stearothermophilus* plasmid pTHT15 (RepB), as well as a chloramphenicol acetyltransferase (*cat*) gene, which was derived from *Staphylococcus aureus* plasmid pC194 and has been used for selections in thermophiles at temperatures of 50–55°C [59], [60].

For gene targeting in *C. thermocellum*, pHK-TT1A plasmids expressing the thermotargetrons were electroporated into wild-type strain DSM 1313, and transformants were selected by plating on GS-2 medium containing thiamphenicol, a derivative of chloramphenicol. In successful experiments, after incubating the plates for 5 days at 51°C, we obtained 1 to 100 thiamphenicol-resistant colonies for each thermotargetron construct, with most constructs giving 20 to 50 transformants. The transformants were then screened for thermotargetron insertion at the desired site by colony PCR and precise insertion was confirmed by sequencing across the 5′- or 3′-integration junction. Targeting efficiencies were calculated as the percentage of transformants containing the insertion.

**Figure 6:**
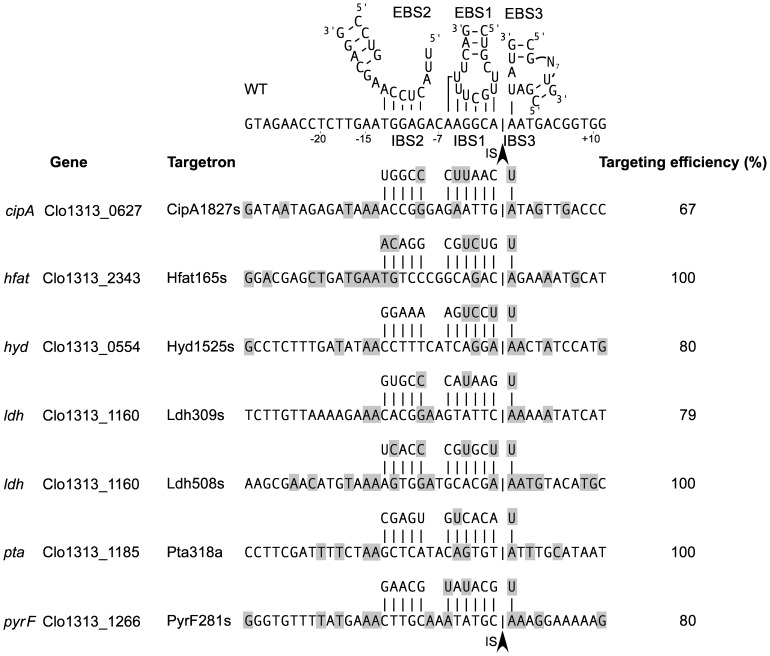
Validated thermotargetrons for *C. thermocellum*. The Figure shows target sites, EBS/IBS base-pairing interactions, and targeting efficiencies for seven targetrons that gave site-specific gene disruptions in *C. thermocellum*. The targeted genes and their gene products were: *cipA* (Clo1313_0627), cellulosome scaffoldin protein; *hfat* (Clo1313_2343), hypothetical formate acetyltransferase; *hyd* (Clo1313_0554), hydrogenase; *ldh* (Clo1313_1160), lactate dehydrogenase; *pta* (Clo1313_1185), phosphotransacetylase; *pyrF* (Clo1313_1266), orotidine 5′-phosphate decarboxylase. Gray shading highlights nucleotide residues in the *C. thermocellum* target sites that match those in the wild-type target site, which is shown above for comparison. The arrowhead indicates the intron-insertion site (IS). The targeting efficiency was calculated as the percentage of thiamphenicol-resistant transformants in which the disruption of the target gene was detected by colony PCR and confirmed by sequencing across the 5′- or 3′-intron integration junction (see Fig. 7).

**Figure 7:**
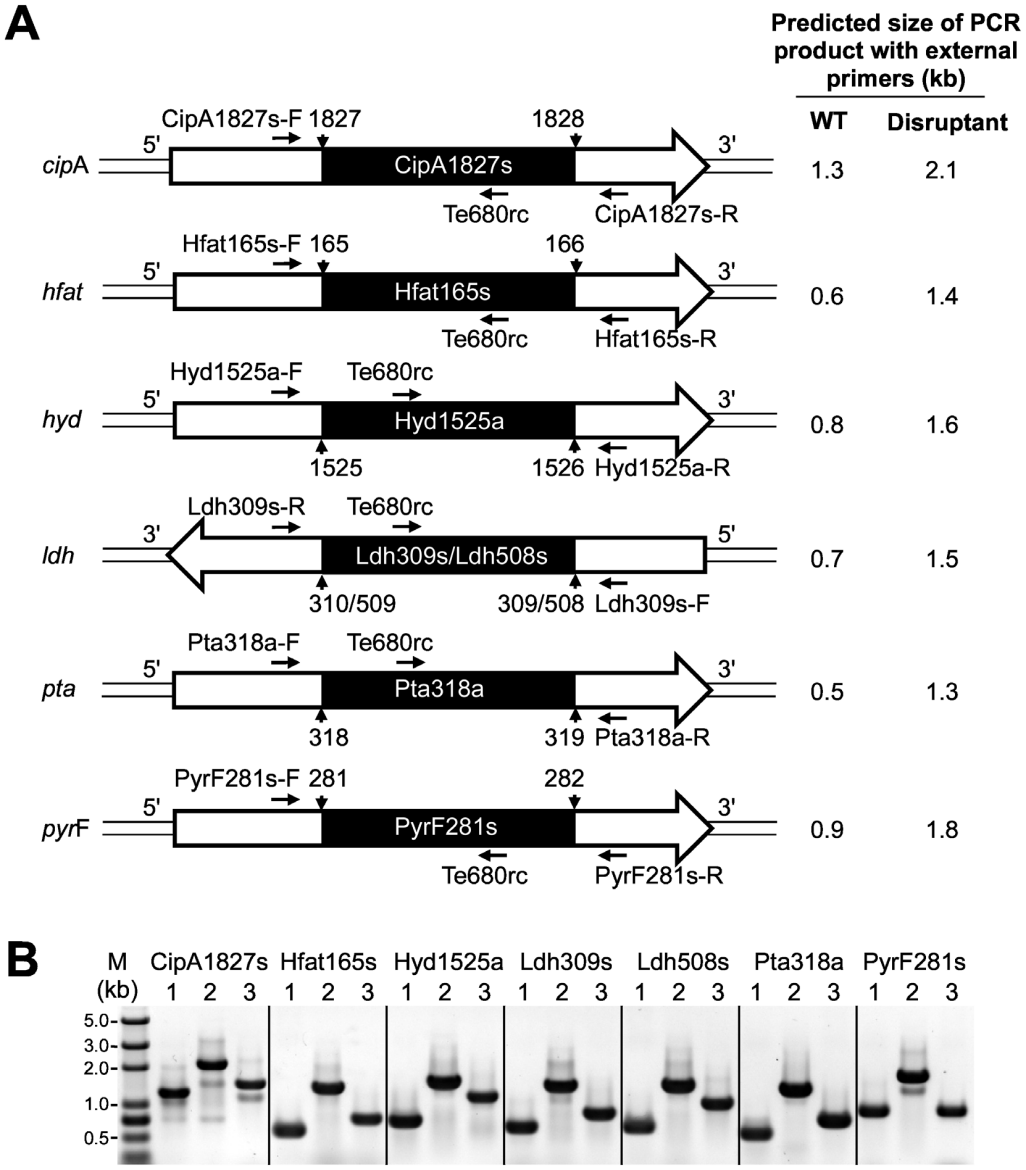
PCR analysis of thermotargetron insertions in chromosomal genes of *C. thermocellum* DSM 1313. (A) Schematic representation of the insertion of seven targetrons into chromosomal genes of *C. thermocellum* DSM 1313. Genomic DNA is indicated by a double line, and the ORF of the target gene is indicated by an open arrow, whose direction indicates whether the ORF is located on the positive (5′ to 3′) or negative (3′ to 5′) DNA strand. Inserted targetrons are indicated by black boxes, with the insertion junctions indicated by arrowheads with nucleotide position numbers in the target gene. PCR-primer binding sites and primer orientations are indicated by horizontal arrows. The binding sites for the external primer sets are located within the target genes upstream or downstream of the targetron-insertion site. The internal primer Te680rc base pairs to the sense strand of the intron (nucleotide positions 658–675; Table S3). The expected sizes (kb) of the PCR products obtained with the external primers for the wild-type (WT) and disrupted genes are indicated to the right. (B) Colony PCR analysis of seven targetron insertions in chromosomal genes. Three PCRs were performed for each targetron. Lane 1, using the external primers and wild-type DNA as the template; lane 2, using the external primers and the disruptant DNA as the template; lane 3, using the external forward or reverse primer and internal primer Te680rc with the mutant DNA as the template; M, DNA markers.

**Figure 8:**
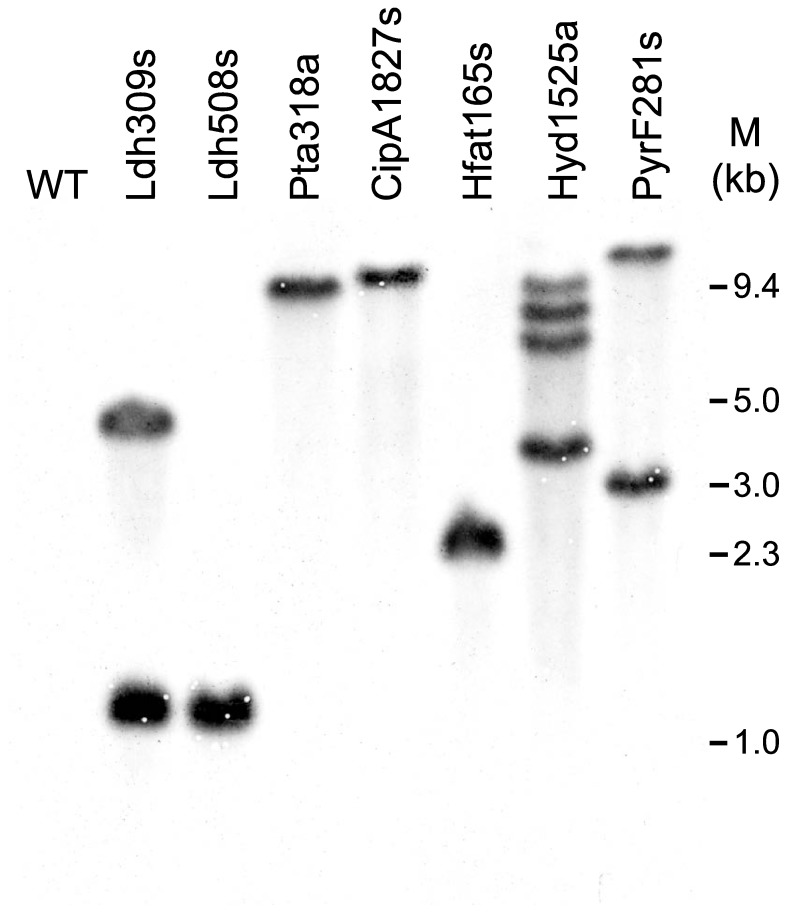
Southern hybridization analysis of thermotargetron insertions in chromosomal genes of *C. thermocellum* DSM 1313. After curing the targetron expression plasmid, genomic DNAs of wild-type or disruptant strains were digested with EcoRI and BamHI, run in a 0.8% agarose gel, and blotted to a Nylon membrane (Hybond-NX, GE Healthcare). The blots were hybridized with a DIG-labeled probe for the TeI3c intron (nucleotide positions 539–710) and visualized by immunological detection according to the manufactor’s protocol (DIG-High Prime DNA Labeling and Detection Starter Kit I, Roche). M, DNA markers.

By using the above procedures, we obtained seven thermotargetrons (CipA1827s, Hfat165s, Hyd1525a, Ldh309s, Ldh508s, Pta318a, and PyrF281s) that inserted into the desired site in six different *C. thermocellum* genes [*cipA* (Clo1313_0627), *hfat* (Clo1313_2343), *hyd* (Clo1313_0554), *ldh* (Clo1313_1160), *pta* (Clo1313_1185), and *pyrF* (Clo1313_1266)] with targeting frequencies ranging from 67 to 100% without selection (Fig. 6). For six of these thermotargetrons (the exception was Hfat165s), the initial colony PCR screening showed bands derived from both the wild-type and disrupted alleles, indicating mixed populations of cells. Thus, the colonies were restreaked on fresh GS-2 solid medium containing thiamphenicol to isolate pure populations of the desired disruptant (Fig. 7). Southern hybridizations after curing the targetron expression plasmid showed that four of the disruptants (those obtained with CipA1827s, Pta318a, Ldh508s and Hfat165s) contained a single thermotargetron insertion at the desired site, but the remaining disruptants (Ldh309s, PyrF281s and Hyd1525a) had one or more additional bands, indicating off-target integrations (Fig. 8). In one case (Ldh508s), it was necessary to restreak multiple times to obtain the desired single disruptant.

We failed to obtain disruptants for 18 additional thermotargetrons that were tested in parallel. In two cases, thermotargetrons targeted to different sites in *gly3* (Clo1313_0396), ∼30 thiamphenocol-resistant transformants were obtained after electroporation of the targetron donor plasmid, but none were found to have the desired disruptions by colony PCR. In the remaining 16 cases [2 targetrons for *ldh* (Clo1313_1878), 1 for *pta* (Clo1313_1185), 2 for *fat* (Clo1313_1717), 2 for *ack* (Clo1313_1186), 1 for *hfat* (Clo1313_2343), 2 for *hyd* (Clo1313_1881), 2 for *hyd* (Clo1313_1791), 2 for *hyd* (Clo1313_0571), 2 for *hyd* (Clo1313_0573), and 1 for *fur* (Clo1313_1691)], we obtained no thiamphenicol-resistant transformants in at least three separate electroporations of the targetron donor plasmid. The failure to obtain thiamphenicol-resistant transformants for these thermotargetrons could reflect the low, variable transformation efficiency of *C. thermocellum* or that the thermotargetron is deleterious, either because of harmful off-target integrations or because the target gene is essential. Hydrogenases, which catalyze the reversible oxidation of molecular hydrogen, play a vital role in anaerobic metabolism by controlling excessive reducing equivalents [61]. Although we constructed thermotargtrons for all five putative hydrogenases genes in *C. thermocellum* DSM 1313, only Hyd1525a targeted to Clo1313_0554 gave disruptants (Fig. 6), and these showed no obvious growth changes compared to the wild-type strain in a preliminary fermentation test with cellobiose as the carbon source, indicating that this gene is not essential. In more recent experiments, we were successful in obtaining thermotargetron disruptions at two additional sites in the *cipA* gene and in disrupting a secondary scaffoldin-encoding gene (Clo1313_1487) in *C. thermocellum* DSM 1313 (unpublished data).

### Fermentation Analysis of *C. thermocellum* with Single and Double Disruptions in the Genes Encoding Lactate Dehydrogenase and Phosphotransacetylase

Ethanol, acetate and lactate are three main fermentation end-products of *C. thermocellum*. Lactate dehydrogenase (Ldh) catalyzes the reduction of pyruvate to lactate, and phosphotransacetylase (Pta) participates in the production of acetate. Metabolic engineering was previously performed in *C. thermocellum* to enhance the production of ethanol by deleting the *ldh* (Clo1313_1160) and *pta* (Clo1313_1185) genes via homologous recombination, requiring complex plasmid constructions, specific selection markers, and laborious screening [59]. Here, we tested how thermotargetron disruption of these genes affects carbon metabolism and ethanol production.

Besides the Ldh mutant (DSM 1313 *ldh*::Ldh309s) and Pta mutant (DSM 1313 *pta*::Pta318a), a double mutant DSM 1313 *ldh*::Ldh309s, *pta*::Pta318a was constructed by introducing thermotargetron Pta318a into the *C. thermocellum* Ldh mutant after curing the plasmid expressing the Ldh targetron. This double disruptant contains an additional off-target integration that was identified by PCR and sequencing as being in Clo1313_2042, which is annotated as encoding a proteinase inhibitor and is not expected to affect carbon metabolism. Growth curves of the wild-type and mutant strains using cellobiose as the carbon source showed that the growth of the Ldh mutant was enhanced, while the Pta mutant and the Ldh Pta double mutant exhibited a prolonged lag phase and depressed growth compared to the wild-type strain (Fig. S1).

**Figure 9:**
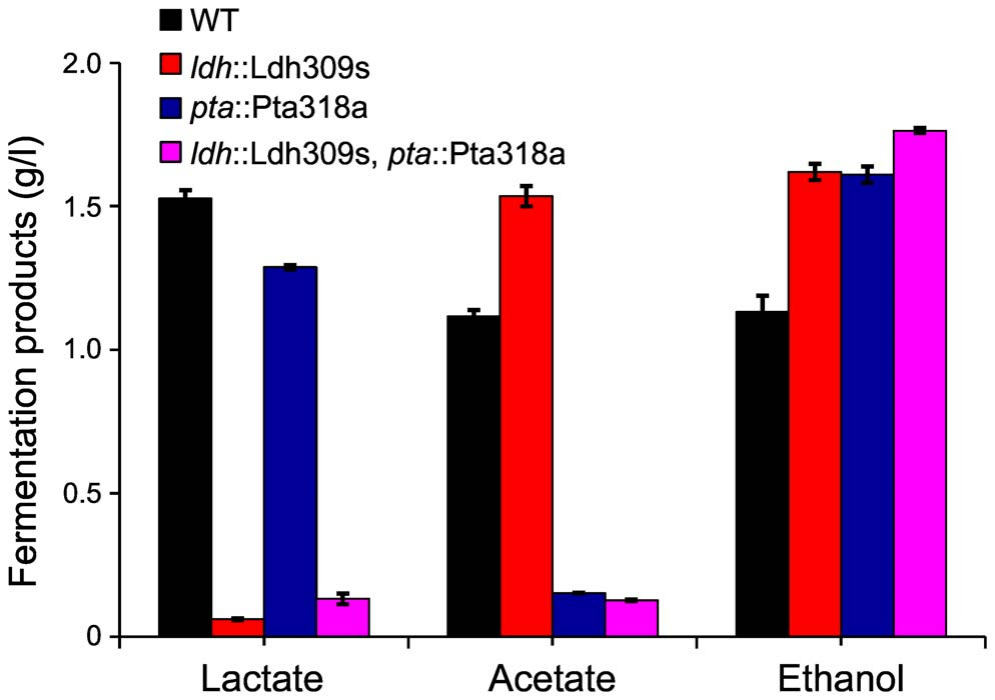
HPLC analysis of extracellular metabolites produced by *C. thermocellum* wild-type DSM 1313 and mutant strains with cellobiose as the sole carbon source. The strains were: WT, *C. thermocellum* wild-type DSM 1313; DSM 1313* ldh*::Ldh309s; DSM 1313 *pta*::Pta318a; and double mutant DSM 1313 *ldh*::Ldh309s, *pta*::Pta318a. The fermentation time was 110 h, and the values are the mean for three independent fermentations with the error bars indicating the standard deviation.

The extracellular metabolites resulting from fermentation by the wild-type and mutant strains after curing of the targetron plasmid were analyzed by HPLC (Fig. 9, Fig. S2). With cellobiose as the carbon source, lactate production by the Ldh disruptant (*ldh*::Ldh309s) was decreased to 4% of the wild-type level, while acetate and ethanol production increased by 37% and 45%, respectively (Fig. 9). By contrast, in the Pta mutant *(pta*::Pta318a), lactate and acetate production were decreased to 81.5 and 13.5% of the wild-type levels, respectively, while ethanol production was increased by 42%. The double mutant showed strong decreases in both lactate and acetate production (8.6 and 11.3% wild-type, respectively), while ethanol production was increased by 56% (Fig. 9). Fermentation with Avicel as the carbon source showed similar patterns of metabolite production, but with smaller percent increases in ethanol production in the mutants (Fig. S2).

**Figure 10:**
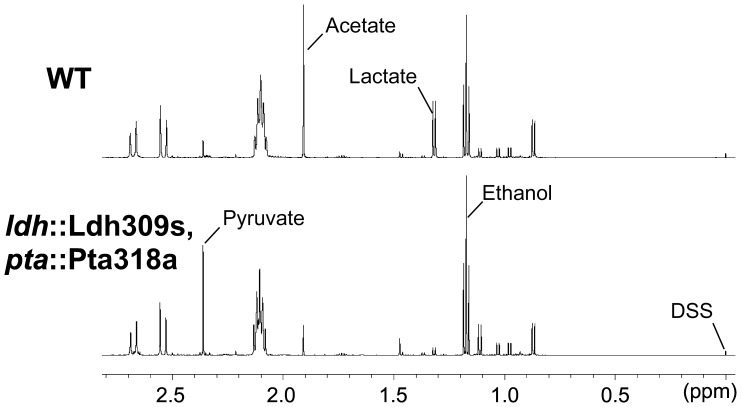
H^1^NMR spectra of the extracellular metabolites of *C. thermocellum* DSM 1313 strains cultured with cellobiose as the sole carbon source. The strains were WT, *C. thermocellum* wild-type DSM 1313 and the double mutant DMS 1313 *ldh*::Ldh309s, *pta*::Pta318a. Peaks for lactate, acetate, ethanol, and pyruvate are marked. The ratios of the integrals of representative metabolite peaks and internal reference (0.5 mM DSS) were used to calculate the metabolite concentrations against standard curves, as described in Materials and Methods. The concentrations of pyruvate produced by the wild-type and double mutant strains were calculated to be 0.73 and 4.12 mM, respectively.

The extracellular metabolites of the wild-type and double mutant strains were further analyzed by nuclear magnetic resonance (NMR) spectroscopy, which showed that pyruvate production of the double mutant was six times higher than that of the wild-type strain (Fig. 10). This finding, which is in agreement with previous results for the *C. thermocellum ldh* and *pta* deletions obtained by homologous recombination [59], suggests that accelerating the carbon flux from pyruvate to ethanol will be required to further enhance ethanol production.

## Discussion

Here, we describe the construction of a thermotargetron derived from a mobile group II intron found in the thermophilic cyanobacterium *T. elongatus*. After determining DNA-target site recognition rules for this thermotargetron in *E. coli* at 48°C, we used it in *C. thermocellum* to disrupt six different chromosomal genes at high efficiency (67–100% without selection). Like mesophilic targetrons, the thermotargetron integrates site-specifically at efficiencies that are high enough to detect by colony PCR without selection, even among a small number of transformants; can be used in RecA^+^ or RecA^−^ bacterial strains; and has a broad host range, with the ability to function in both Gram-negative (*E. coli*, *T. elongatus*) and Gram-positive (*C. thermocellum*) bacteria. Thus, we anticipate that it will be useful for gene targeting in a variety of thermophiles, as well as mesophiles that can tolerate short periods at elevated temperatures.

For use in *C. thermocellum*, we constructed a thermotargetron expression cassette that uses the promoter of the *C. thermocellum groEL* gene to express the thermotargetron group II intron RNA and RT and cloned it to an *E. coli*/*C. thermocellum* shuttle vector derived from pNW33N. The resulting thermotargetron expression vector, denoted pHK-TT1A, contains a ColE1 replication origin that functions in Gram-negative bacteria, a RepB replication origin that functions in thermophilic Gram-positive bacteria, and a chloramphenicol-resistance gene from *S. aureus* plasmid pC194, which has been used previously for selection in thermophiles at 50–55°C [59], [60] (Fig. 5). The very high targeting efficiencies of the thermotargetron make it possible to identify disruptants by colony PCR without including a selectable marker in the intron, thereby facilitating the construction of strains with multiple gene disruptions. In principle, thermotargetrons could also be used to site-specifically insert cargo genes cloned within the intron, although such insertions decrease targeting efficiency, sometimes substantially [27], [62]. In mesophiles, targetrons can be used to site-specifically position short recombinase sites (*e.g.*, Cre/LoxP) that can then be used to integrate separately transformed DNAs by recombination (http://www.sigmaaldrich.com/targetron), and a future step will be to construct such a system for thermophiles.

The mobile group II intron that we used to construct the thermotargetron evolved to retrohome in *T. elongatus*, an organism that has an optimal growth temperature of 50–60°C [63]. Here, we find that the thermotargetron is active in *E. coli* at temperatures >42°C with activity increasing with increasing temperature up to 48°C, above which cells lose viability. Gene targeting in *C. thermocellum* was done at 51°C, but further experiments showed that the thermotargetron remains active in *C. thermocellum* at higher temperatures (tested up to 65°C; unpublished data). Both the intron RNA and IEP components of the thermotargetron evolved to function at high temperature, and elsewhere we show that the TeI4c IEP has thermostable RT activity that is capable of synthesizing cDNAs at temperatures up to 81°C [64].

Our success rate in obtaining targeted gene disruptions in *C. thermocellum* was 7 of 25 targetron constructs tested. In most cases, however, the failures were due to inability to obtain thiamphenicol-resistant transformants after electroporation of the targetron donor plasmid. In addition to inefficient transformation, such failures could reflect that the target genes are essential or that these thermotargetrons gave deleterious off-target integrations. In the future, the proportion of successful thermotargetrons may be improved by: (i) incorporating genetic markers, including RAM markers constructed for thermophiles, enabling the detection of disruptions by less efficient thermotargetrons; (ii) the further refinement of DNA targeting rules for base-pairing interactions, which assume greater importance for thermotargetron at higher temperatures; and (iii) selecting targetrons that have the most unique integration sites in the *C. thermocellum* genome to minimize the possibility of off-target integrations (see below). The seven targetrons validated here could now be used to obtain the same disruptions in any strain of *C. thermocellum* in which the target site is sufficiently conserved.

Like mesophilic targetrons, the thermotargetron recognizes DNA target sequences by using both the IEP and base pairing of the intron RNA, with the latter providing most of the DNA target specificity. Thermotargetron differs, however, in that the number of nucleobases recognized by the IEP is smaller than commonly found for mesophilic targetrons. This difference appears to reflect that the thermotargetron operates at high temperatures that help promote DNA melting and is thus less dependent upon energy derived from IEP binding for DNA strand separation [55]. The more limited protein recognition increases the number of potential insertion sites, thereby increasing the number of thermotargetrons that can be tested for each target gene. The *E. coli lacZ* gene, for example, contains 160 potential insertion sites that match the short IEP recognition sequence for thermotargetron (WAA; see Results), compared to 13 and 5 target sites that match the five nucleotide residues most stringently recognized by the IEP for the Ll.LtrB and EcI5 targetrons (Ll.LtrB: −21G, −20A, −19T, −17A, and +5T; EcI5: −18C, −17C, −15A, −14A, and +5T [25], [28]). The more relaxed protein recognition of thermotargetron should facilitate the targeting of short ORFs and small non-coding RNAs not only in thermophiles, but also in mesophiles at moderately elevated temperature.

A drawback of more limited IEP recognition by thermotargetron is that it decreases target specificity leading to a greater number of off-target integrations than are typically observed for mesophilic targetrons. Despite this drawback, we could obtain single insertions by further restreaking and rescreening when attempted in the majority of cases. Further precautions and improvements that may decease off-target integrations include the use of a more readily curable donor plasmid and/or an inducible promoter to avoid continuous targetron expression, and scanning the host genome for close matches to potential target sites, which was not done for the thermotargetrons tested here.

Because *C. thermocellum* is a promising candidate for CBP production of cellulosic ethanol, we demonstrated the application of thermotargetron in this organism by targeting chromosomal genes that play important roles in cellulose utilization and metabolism (Fig. 6). For example, *cipA* encodes a major scaffoldin protein of the cellulosome, a multi-enzyme complex that functions in cellulose degradation; the *hyd* genes encode hydrogenases, which are important in maintaining redox balance; and *hfat* encodes a putative formate acetyltransferase, which may participate in formate production from pyruvate, the major intermediate in the ethanol producing pathway. We then focused on the *ldh* and *pta* genes, which encode enzymes involved in the production of lactate and acetate, respectively, the major by-products of cellulosic ethanol production in *C. thermocellum*. Fermentation analysis showed that the disruption of either *ldh* or *pta* by thermotargetrons in *C. thermocellum* strain DSM 1313 increased ethanol production by 37 and 42%, respectively. Although the double mutant showed strong decreases in both lactate and acetate production, its ethanol production was increased by only 56% (Fig. 9, Fig. S2), while pyruvate production measured by NMR was increased by six-fold (Fig. 10). These results are consistent with previous analysis of *ldh* and *pta* deletions obtained by homologous recombination in *C. thermocellum* strain DSM 1313 [59] and suggest that additional genetic engineering of pyruvate metabolism will be needed to further increase ethanol production. In addition to *C. thermocellum*, a variety of other thermophiles have been used as microbial factories for the production of chemicals or thermostable proteins [65], [66]. Given its broad host range, we anticipate that thermotargetron will be generally useful for increasing the efficiency of chemical and protein production in these organisms.

## Materials and Methods

### Bacterial Strains and Growth Conditions

*E. coli* HMS174(DE3) (Novagen) was used for retrohoming assays and DH5α (Life Technologies) was used for cloning (Table S1). Strains were grown in Luria-Bertani (LB) medium with shaking at 200 rpm under conditions described for individual experiments. Antibiotics were added at the following concentrations when needed: ampicillin, 100 µg/ml; chloramphenicol, 25–50 µg/ml; tetracycline, 25 µg/ml.

*C. thermocellum* DSM 1313 (Table S1) was cultured at 55°C anaerobically in modified GS-2 medium (KH_2_PO_4_ 1.5 g, K_2_HPO_4_·3H_2_O 2.1 g, urea 2.1 g, MgCl_2_·6H_2_O 1.0 g, CaCl_2_·2H_2_O 150 mg, FeSO_4_·6H_2_O 1.25 mg, cysteine-HCl 1 g, MOPS-Na 10 g, yeast extract 6.0 g, trisodium citrate·2H_2_O 3.0 g, resazurin 0.1 mg per liter, pH 7.4) [57], unless otherwise noted. Cellobiose (6–10 g/l) or Avicel (10 g/l) were used as the carbon source. 0.8% agar was added for solid medium, and thiamphenicol was added at 3–6 µg/ml, when needed. All media were purged with high purity nitrogen gas for at least 5 min to maintain anoxic conditions.

### Recombinant Plasmids

The plasmids used in this study are listed in Table S2. The intron-donor plasmids pACD2X-TeI4h*/4h* and pACD2X-TeI3c/4c and the recipient plasmids pBRR-3c and pBRR-4h used for retrohoming assays in *E. coli* were described previously [55]. Recipient plasmids containing different DNA target sites were constructed by swapping in a synthetic double-stranded DNA oligonucleotide containing target site positions −30 to +15 between the PstI and EcoRI sites of pBRR-tet, as described [13], [55].

The thermotargetron donor plasmid pACD2-TT1A was derived from pACD2X-TeI3c/4c by introducing an SpeI site upstream of IBS2 in the 5′ exon and a BsiWI site between EBS1 and EBS3 within the intron, thereby enabling the swapping in of a short (357-bp) SpeI+BsiWI fragment containing retargeted IBS1 and 2 and EBS1 and 2 sequences. It was constructed in two steps via PCRs with primers that introduce the mutations. First, the mutation T-20A was introduced into the 5′ exon to create the SpeI site and then the mutations T319A, A321G, T337C, and A339T were introduced into the intron to create the BsiWI site and maintain base pairing in stem ID(ii) (Fig. 1A). Finally, the T7 promoter sequence in DIV was deleted by replacing the 516-bp BsiWI+PstI fragment with one generated by PCR from the native TeI3c intron cloned in pUC18 [55].

pACD2-TT1C, G, and T are derivatives of pACD2-TT1A that have the indicated nucleotide residue at IBS3 in the 3′ exon and the complementary nucleotide residue at EBS3 in the intron to maintain the EBS3/IBS3 pairing in the unspliced precursor RNA. These additions enable targeting of DNA sites with the indicated nucleotide residues at the IBS3 position. The plasmids were constructed by PCR of pACD2X-TeI3c/4c with primers TeI3cEBS3mutA, C, or G and TeI3cIBS3T, G or C-3Pst that introduce the EBS3 and IBS3 changes, respectively (Table S3). The resulting PCR products were digested with BsiWI and PstI and swapped for the corresponding fragment of pADC2-TT1A.

Plasmid pIKM1-TT1A contains the *C. thermocellum groEL* promoter followed by the TeI3c intron/TeI4c RT cassette from pACD2-TT1A cloned between the BamHI and EcoRI sites of the vector pIKM1 [67]. pIKM1 contains a thermostable *kan^R^* marker, a gram-negative ColE1 replication origin, an *amp^R^* marker, a Gram-positive pIM13 (ORF2) origin, and a MLS (Macrolide Lincosamine Streptogramin) marker [67]. pIKM1-TT1A was constructed in two cloning steps. In the first step, the *C. thermocellum groEL* promoter was amplified from plasmid pJIR750ai_GroEL_promoter-CelS [58] by PCR with primers Ct_PgroEL5, which introduces a BamHI site, and Ct_PgroEL3, which introduces SpeI, XhoI and EcoRI sites (Table S3), and the resulting PCR product was digested with EcoRI and BamHI and cloned between the corresponding sites of pIKM1 to generate the intermediate plasmid pIMK1PgroEL. In the second step, the TeI3c/TeI4c cassette from pADC2-TT1A was reconstituted from two gel-purified DNA fragments (a 897-nt SpeI/PstI fragment containing TeI3c and a 1716-nt PstI/XhoI fragment containing the TeI4c RT ORF) and cloned downstream of the GroEL promoter in pIMK1PgroEL via a three-fragment ligation.

Plasmid pHK-TT1A, used for thermotargetron expression in *C. thermocellum*, is a derivative of pNW33N (Genbank AY237122; BGSC). To minimize the size of the final plasmid [12], a 760-bp fragment of pNW33N between the *E. coli* replication origin (ColE1) and chloramphenicol-resistance gene (*cat*) of pNW33N was deleted by reverse PCR of pNW33N using primers Phk-F and Phk-R (Table S3), followed by digestion with EcoRI and self-ligation. The resulting plasmid, denoted pHK, contains a multiple cloning site region with eleven single restriction sites (PstI, NarI, SmaI, BamHI, XhoI, EcoRI, XbaI, HindIII, KpnI, NdeI, and NheI) in place of the deleted DNA segment. To generate the thermotargetron expression plasmid pHK-TT1A, pIMK1-TT1A (see above) was digested with EcoRI and BamHI, and the 2.8-kb fragment containing the *groEL* promoter and Tel3c/4c targetron cassette was cloned between the EcoRI and BamHI sites of pHK. Except for the CipA1827s and pyrF281s thermotargetrons, which were transferred by cloning EcoRI+BamHI fragments of pIMK1P-TT1A into pHK, as described above, thermotargetrons were constructed directly in pHK-TT1A by replacing the 357-bp SpeI+BsiWI fragment with one containing modified IBS1, IBS2, EBS1, and EBS2 sequences generated by SOEing PCRs [68] (see below).

### Targeting of Thermotargetron to Desired Sites

Thermotargetrons are targeted to insert into desired sites by: (i) searching the DNA target sequence for matches to the sequence WAA (where W is A or T) at positions −16 to −14 from the intron-insertion site; (ii) generating a 357-bp PCR product in which the EBS1 and EBS2 sequence in the intron RNA are modified to base pair to IBS1 positions −1 to −6 and IBS2 positions −8 to −13 of the DNA target site, and the IBS1 and IBS2 sequences in the 5′ exon of the donor plasmid are modified to base pair to the retargeted EBS1 and EBS2 sequences for efficient RNA splicing; and (iii) swapping the PCR product containing the modified IBS1 and 2 and EBS1 and 2 sequences into one of four different targetron donor plasmids that enable recognition of different nucleotide residues at IBS3 (position +1). The 357-bp PCR product with retargeted EBS1, EBS2, IBS1, and IBS2 sequences was generated in two PCR steps. In the first step, two PCRs were done with overlapping primers to amplify two overlapping segments of the intron. The upstream segment was amplified with a 5′ primer that changes IBS1 and IBS2 and has a 5′ terminal SpeI site (primers denoted xxxxIBS1/2, where xxxx indicates the target gene and the position of the insertion site in the bottom/antisense (“a”) or sense/top (“s”) strands), and a universal 3′ primer (TeI3cUNI) that is complementary to the 5′ primer of the second PCR. The downstream segment was amplified with a 5′ primer (xxxxEBS2) that is partially complementary to the universal primer and changes EBS2, and a 3′ primer (xxxxEBS1a) that changes the EBS1 sequence and has a 5′ terminal BsiWI site (Table S3). In the second step, the two PCR products were gel-purified and used in a second PCR with the xxxxIBS1/2 and xxxxEBS1a outside primers to generate a 357-bp product with the modified IBS1, IBS2, EBS1, and EBS2 sequences and terminal SpeI and BsiWI sites that was then swapped for the corresponding segment of the targetron expression plasmid. The resulting targetrons are denoted by a number that corresponds to the nucleotide residue 5′ to the intron-insertion site within the target gene, followed by “a” or “s” indicating the antisense/bottom or sense/top strands, respectively.

### Intron Retrohoming Assays and Gene Targeting Experiments in *E. coli*

For intron retrohoming assays, *E. coli* HMS174(DE3) was co-transformed with the Cap^R^-donor and Amp^R^-recipient plasmids, inoculated into 5 ml of LB medium containing ampicillin and chloramphenicol, and grown overnight with shaking (200 rpm) at 37°C. A small portion (50 µl) of the overnight culture was inoculated into 5-ml of fresh LB containing the same antibiotics and grown for 1 h as above. The cells were then induced by adding 1 ml of additional LB containing ampicillin, chloramphenicol and 3 mM IPTG (500 µM final) and incubating for times and at temperatures indicated in Figures for individual experiments. After induction, the culture was placed on ice, diluted with ice-cold LB, plated at different dilutions onto LB agar medium containing ampicillin or ampicillin+tetracycline, and incubated overnight at 37°C. Retrohoming efficiencies in these plasmid-based assays were quantified as the ratio of (Tet^R^+Amp^R^)/Amp^R^ colonies. For determination of temperature dependence, the initial 5-ml log-phase cultures grown at 37°C were mixed with an equal volume of fresh LB medium containing antibiotics and 1 mM IPTG (500 µM final) that had been pre-warmed to achieve the desired temperature.

For targeting of the *E. coli lacZ* gene, donor plasmids expressing the retargeted intron were transformed into *E. coli* HMS174(DE3) and grown overnight in LB medium containing chloramphenicol. An aliquot of the overnight culture was diluted 100-fold into fresh LB supplemented with chloramphenicol, incubated for 1 h at 37°C, and induced with 500 µM IPTG at 48°C for times specified in figure legends for individual experiments. Cells were plated at different dilutions onto LB+X-Gal agar, and the plates were incubated overnight at 37°C for counting of blue and white colonies. Colonies were picked, restreaked and analyzed by colony PCR using primers that amplify the region of the *lacZ* gene containing the targetron insert (primers lacZ30s+lacZ1850a or lacZ1850s+lacZ3060a) or the 5′- and 3′-integration junctions (primers lacZ1850a or lacZ3060a+TeI3c680rc and lacZ30s or lacZ1850s+TeI3c420s, respectively) (Table S3). The targetron-insertion sites were confirmed by sequencing the PCR products with either the Te420f or Te680rc primers (Table S3). For Southern hybridizations, strains were cured of the targetron expression plasmid, and chromosomal DNA was isolated by the CTAB/NaCl method [69], digested with ApaI and EcoRI, run in a 0.9% agarose gel, blotted to Nylon membrane (Hybond-NX, GE Healthcare), and hybridized with ^32^P-labeled DNA probes (High prime labeling kit, Roche) for the *lacZ* gene (nucleotides 30–1850) or the TeI3c intron (nucleotides 1–342), as described [28]. The blots were scanned with a Typhoon Trio PhosphorImager (GE Healthcare).

### Use of Thermotargetron for Gene Targeting in *Clostridium thermocellum*

Transformation of *C. thermocellum* DSM 1313 was performed according to the reported protocol in an anaerobic chamber with slight modifications [11], [12]. *C. thermocellum* competent cells were prepared by cultivation at 55°C anaerobically with cellobiose (5 g/l) as the carbon source until the O.D._600_ was 0.5–0.8. Cells were collected by centrifugation (4°C, 2500×g, 10 min) and washed twice in 15% ice cold, sterile, and oxygen-free glycerol. 50 µl of the cell suspension was added to a 0.1-cm electroporation cuvettete (BioRad) with 1–10 µl of DNA (10–2,000 ng) in sterile distilled water. A series of 40 square pulses was applied, each with an amplitude of 1.5 kV and duration of 50 µs at 500 µs intervals. After electroporation, cells were allowed to recover for 15–20 h at 51°C in 4 ml of antibiotic-free GS-2 medium, and then plated on solid medium containing thiamphenicol (Tm) at a final concentration of 3–6 µg/ml. The plates were incubated at 51°C for 5 days, then colonies were picked and inoculated into 4 ml of fresh GS-2 medium supplemented with thiamphenicol. A portion of the cell suspension was used for colony PCRs to screen for targetron insertions in the desired genes. Colony PCR was done with forward and reverse primers flanking the target gene to check for full-length (0.8-kb) targetron insertion and with an internal primer (Te680rc) and the flanking forward or reverse primer to PCR across the 5′- or 3′-integration junction, respectively (Figure 7, Table S3). The integration junctions were verified by sequencing.

Targetron expression plasmids were cured by growing cells in the absence of antibiotic. A 10-µl portion of a cell suspension was inoculated into 4 ml of fresh GS-2 medium without thiamphenicol and incubated at 51°C for 2 days. Then 500 µl of the culture was inoculated into 4 ml of fresh GS-2 medium containing thiamphenicol, and curing of the plasmid was verified by inability of the cells to grow in the presence of the antibiotic. The process was repeated once or twice as needed to cure the plasmid.

Southern hybridization to check the targetron insertion in *C. thermocellum* chromosomal DNA was performed as described [70], after curing the targetron expression plasmid. To isolate genomic DNAs for Southern hybridizations, wild-type and mutant cells were cultivated at 51°C in 5 ml GS-2 medium with cellobiose as carbon source until late exponential phase (O.D._600_ ≈ 1.0), and then collected by centrifugation at 5000×g for 5 min. Genomic DNA was isolated by using a Bacterial Mini Preparation Kit (BioMed technology) and digested with BamHI and EcoRI at 37°C overnight. The digests were run in a 0.8% agarose gel at low voltage and blotted to a Nylon membrane (Hybond-NX, GE Healthcare). The blots were hybridized with DIG-labeled TeI3c intron probe (nucleotides 539–710) generated by PCR of TeI3c with primers Probe172-F and Probe172-R and visualized by immunological detection according to the manufacturer’s protocol (DIG-High Prime DNA Labeling and Detection Starter Kit I, Roche).

### Fermentation Analysis via HPLC and NMR

*C. thermocellum* strains were incubated at 55°C in 100 ml GS-2 medium anaerobically with cellobiose or Avicel (10 g/l) as the sole carbon source for 110–120 h. Samples were taken every 5 to 10 h with a 2.5-ml syringe, and O.D._600_ was measured immediately with a UV-VIS spectrophotometer. At the end point, samples were centrifuged (12 000×g, 5 min), and the supernatants were micro-filtered (0.22-µm pore diameter) and used as extracellular metabolites samples for analysis of fermentation products. Both high performance liquid chromatography (HPLC) and nuclear magnetic resonance (NMR) were employed to analyze extracellular compounds, including cellobiose, lactate, acetate, pyruvate and ethanol. 20 µl of extracellular metabolites samples were analyzed by HPLC (Agilent 1200 series, Agilent Technologies) equipped with an Aminex HPX-87H column (Bio-Rad) and a refractive index detector (Agilent 1260 infinity RID). 5 mM H_2_SO_4_ was used as the mobile phase at 55°C with a flow rate of 0.5 ml per min [5]. For NMR, 450 µl of sample was mixed with 50 µl of D_2_O, which contained 5 mM 4,4-dimethyl-4-silapentane-1-sulfonic acid (DSS) as an internal reference, and transferred into a 5-mm NMR tube for NMR analysis using a Bruker AVIII 600 MHz NMR spectrometer equipped with a 5-mm cryogenic probe (Bruker Biospin GmbH). Standard 1D ^1^H NMR spectra were recorded and processed using TopSpin software (Bruker Biospin GmbH). Metabolite peaks were assigned by the chemical shifts from Madison-Qingdao Metabolomics Consortium Database [MMCD, http://mmcd.nmrfam.wisc.edu/ [71]]. Metabolite standards (0.01 to 2 g/l) were prepared for both HPLC and NMR analyses. The concentrations of metabolites were calculated based on corresponding standard curves.

## Supporting Information

Figure S1 **Growth curves of** ***C. thermocellum*** **wild-type DSM 1313 and mutant strains with cellobiose as the carbon source.** The strains were: WT, *C. thermocellum* wild-type DSM 1313; DSM 1313* ldh*::Ldh309s; DSM 1313 *pta*::Pta318a; and double mutant DSM 1313 *ldh*::Ldh309s, *pta*::Pta318a. The error bars show standard deviations based on three independent experiments.
(TIF)

Figure S2 **HPLC analysis of extracellular metabolites produced by** ***C. thermocellum*** **wild-type DSM 1313 and mutant strains with Avicel as the sole carbon source.** The strains were: WT, *C. thermocellum* wild-type DSM 1313; DSM 1313* ldh*::Ldh309s; DSM 1313 *pta*::Pta318a; and double mutant DSM 1313 *ldh*::Ldh309s, *pta*::Pta318a. The fermentation time was 120 h, and the values are the mean for three independent fermentations with the error bars indicating the standard deviation.
(TIF)

Table S1 **Bacterial strains used in this study.**
(DOCX)

Table S2 **Plasmids used in this study.**
(DOCX)

Table S3 **DNA oligonucleotides used in this study.**
(DOCX)
