# Supplementary figures and images for: A Targetron System for Gene Targeting in Thermophiles and Its Application in Clostridium thermocellum
Source: PLoS One. 2013 Jul 9;8(7):e69032. doi: 10.1371/journal.pone.0069032 (PMC3706431; doi:10.1371/journal.pone.0069032)

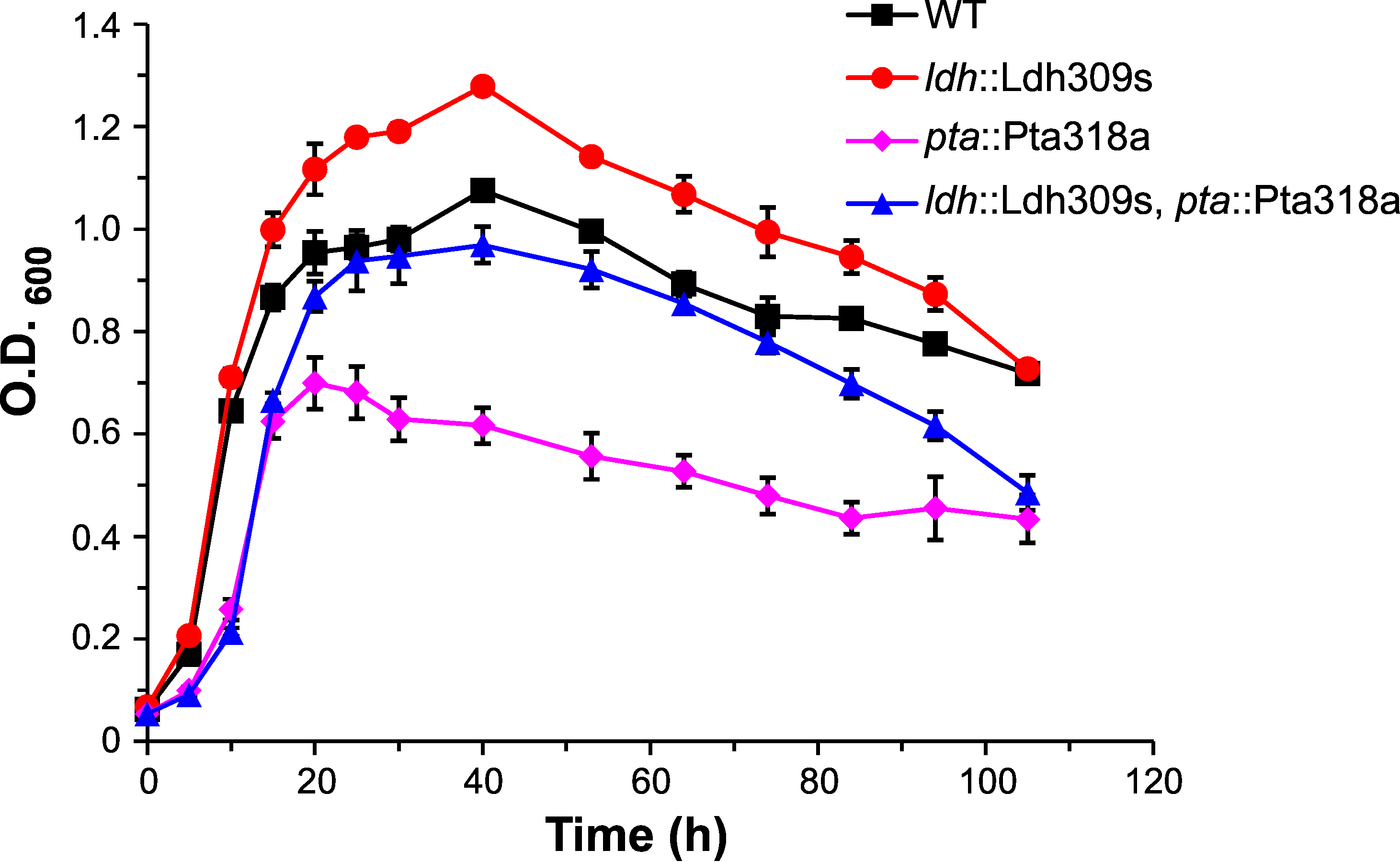

Supplement: Figure S1 — Growth curves of C. thermocellum wild-type DSM 1313 and mutant strains with cellobiose as the carbon source. The strains were: WT, C. thermocellum wild-type DSM 1313; DSM 1313 ldh::Ldh309s; DSM 1313 pta::Pta318a; and double mutant DSM 1313 ldh::Ldh309s, pta::Pta318a. The error bars show standard deviations based on three independent experiments. (TIF) [file pone.0069032.s001.tif]

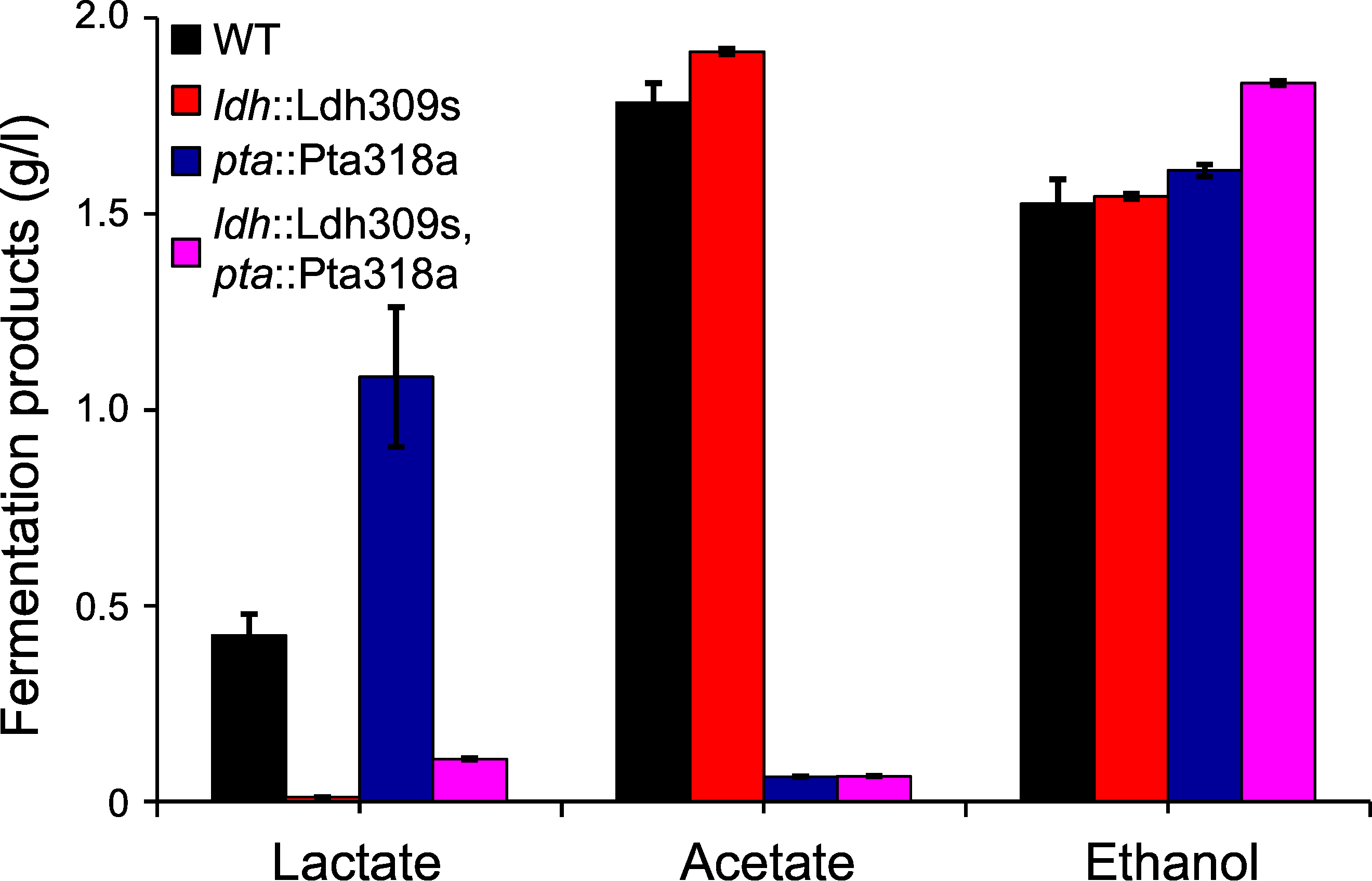

Supplement: Figure S2 — HPLC analysis of extracellular metabolites produced by C. thermocellum wild-type DSM 1313 and mutant strains with Avicel as the sole carbon source. The strains were: WT, C. thermocellum wild-type DSM 1313; DSM 1313 ldh::Ldh309s; DSM 1313 pta::Pta318a; and double mutant DSM 1313 ldh::Ldh309s, pta::Pta318a. The fermentation time was 120 h, and the values are the mean for three independent fermentations with the error bars indicating the standard deviation. (TIF) [file pone.0069032.s002.tif]
