# Supplementary material for: A Targetron System for Gene Targeting in Thermophiles and Its Application in Clostridium thermocellum
Source: PLoS One. 2013 Jul 9;8(7):e69032. doi: 10.1371/journal.pone.0069032 (PMC3706431; doi:10.1371/journal.pone.0069032)
Supplement: Table S1 — Bacterial strains used in this study. (DOCX) [file pone.0069032.s003.docx]

**Table S1.** Bacterial strains used in this study.

| **Strain** | **Characteristics** | **Reference or source** |
| --- | --- | --- |
| ***E. coli*** |  |  |
| DH5α | *f80dlacZΔM15 Δ(lacZYA-argF)U169 deoR recA1 endA1 hsdR17(r_k_^-^, m_k_^+^) phoA supE44 l^-^ thi-1 gyrA96 relA1* | Biomed |
| HMS174(DE3)  HMS174(DE3) *lacZ*::LacZ60a  HMS174(DE3) *lacZ*::LacZ369a  HMS174(DE3) *lacZ*::LacZ2586a | F^-^ *recA1 hsdR*(r_K12_^-^ m_K12_^+^) (DE3 [*lacI* lacUV5-T7 gene 1 *ind1* *sam7* *nin5*]) (Rif ^R^)  Derived from HMS174(DE3), *lacZ*::TeI3cLacZ60a  Derived from HMS174(DE3), *lacZ*::TeI3cLacZ369a  Derived from HMS174(DE3), *lacZ*::TeI3cLacZ2586a | Novagen, Madison, WI  This work  This work  This work |
| ***C. thermocellum*** |  |  |
| DSM1313 | Wild-type stain | DSMZ |
| DSM1313 *cipA*::CipA1827s | Derived from DSM1313, Clo1313_0627::TeI3cCipA1827s | This work |
| DSM1313 *hfat*::Hfat165s  DSM1313 *hyd*::Hyd1525a | Derived from DSM1313, Clo1313_2343::TeI3cHfat165s  Derived from DSM1313, Clo1313_0554::TeI3cHyd1525a | This work  This work |
| DSM1313 *ldh*::Ldh309s | Derived from DSM1313, Clo1313_1160::TeI3cLdh309s | This work |
| DSM1313 *ldh*::Ldh508s | Derived from DSM1313, Clo1313_1160::TeI3cLdh508s | This work |
| DSM1313 *ldh*::Ldh309s, *pta*::Pta318a  DSM1313 *pta*::Pta318a  DSM1313 *pyrF*::PyrF281s | Derived from DSM1313, Clo1313_1160::TeI3cLdh309s, Clo1313_1185::TeI3cPta318a  Derived from DSM1313, Clo1313_1185::TeI3cPta318a  Derived from DSM1313, Clo1313_1266::TeI3cPyrF281s | This work  This work  This work |
