# Supplementary material for: A Targetron System for Gene Targeting in Thermophiles and Its Application in Clostridium thermocellum
Source: PLoS One. 2013 Jul 9;8(7):e69032. doi: 10.1371/journal.pone.0069032 (PMC3706431; doi:10.1371/journal.pone.0069032)
Supplement: Table S2 — Plasmids used in this study. (DOCX) [file pone.0069032.s004.docx]

**Table S2.** Plasmids used in this study.

| **Plasmid** | **Characteristics** | **Reference or source** |
| --- | --- | --- |
|  |  |  |
| pACD2X  pADC2X-TeI3c/4c  pADC2X-TeI4h*/4h*  pACD2X-TT1A  pACD2X-TT1C  pACD2X-TT1G  pACD2X-TT1T  pACD2X-TT1A-LacZ60a  pACD2X-TT1A-LacZ369a  pACD2X-TT1A-LacZ2586a  pACD2X-TT1A-CipA1827s  pACD2X-TT1A-PyrF281s  pBRR-tet  pBRR-3c  pBRR-4h  pBRR-3cC  pBRR-3cG  pBRR-3cT  pBRR-LacZ60a  pBRR-LacZ369a  pBRR-LacZ2586a  pBRR-CipA1827s  pBRR-PyrF281s | Ll.LtrB-ΔORF/LtrA intron donor plasmid used in mobility assays, Cm^R^  Derived from pADC2X, contains TeI3c and TeI4c RT instead of Ll.LtrB-ΔORF/LtrA, used in mobility assays, Cm^R^  Derived from pADC2X, contains TeI4h* and TeI4h* RT instead of Ll.LtrB-ΔORF/LtrA, used in mobility assays, Cm^R^  Derived from pADC2XTeI3c/4c, contains SpeI and BsiWI sites, Cm^R^  Derived from pADC2X-TT1A, Tel3c EBS3 T to G mutant, Cm^R^  Derived from pADC2X-TT1A, Tel3c EBS3 T to C mutant, Cm^R^  Derived from pADC2X-TT1A, Tel3c EBS3 T to A mutant, Cm^R^  Derived from pADC2X-TT1A, Tel3cLacZ60a intron, Cm^R^  Derived from pADC2X-TT1A, Tel3cLacZ369a intron, Cm^R^  Derived from pADC2X-TT1A, Tel3cLacZ2586a intron, Cm^R^  Derived from pADC2X-TT1A, Tel3cCipA1827s intron, Cm^R^  Derived from pADC2X-TT1A, Tel3cPyrF281s intron, Cm^R^  Recipient plasmid for mobility assays, Amp^R^  Derived from pBRR-tet, contains the TeI3c target site, Amp^R^  Derived from pBRR-tet, contains the TeI4h target site, Amp^R^  Derived from pBRR-3c, IBS3 A to C mutant, Amp^R^  Derived from pBRR-3c, IBS3 A to G mutant, Amp^R^  Derived from pBRR-3c, IBS3 A to T mutant, Amp^R^  Derived from pBRR-tet, LacZ60a target site, Amp^R^  Derived from pBRR-tet, LacZ369a target site, Amp^R^  Derived from pBRR-tet, LacZ2586a target site, Amp^R^  Derived from pBRR-tet, CipA1827s target site, Amp^R^  Derived from pBRR-tet, PyrF281s target site, Amp^R^ | [1]  [2]  [2]  This work  This work  This work  This work  This work  This work  This work  This work  This work  [3]  [2]  [2]  This work  This work  This work  This work  This work  This work  This work  This work |
| pHK | Derived from pNW33N, engineered multiple cloning site, *E. coli-C. thermocellum* shuttle vector, Cm^R^/Tm^R^ | This work |
| pHK-TT1A | Derived from pHK, *E. coli-C. thermocellum* shuttle vector, *groEL* promoter, Tel3c/TeI4c RT targetron cassette, Cm^R^/Tm^R^ | This work |
| pHK-TT1A-CipA1827s | Derived from pHK, *E. coli-C. thermocellum* shuttle vector, *groEL* promoter, Tel3cCipA1827s intron, Cm^R^/Tm^R^ | This work |
| pHK-TT1A-Hfat165s | Derived from pHK-TT1A, *E. coli-C. thermocellum* shuttle vector, *groEL* promoter, Tel3cHfat165s intron, Cm^R^/Tm^R^ | This work |
| pHK-TT1A-Hyd1525a | Derived from pHK-TT1A, *E. coli-C. thermocellum* shuttle vector, *groEL* promoter, Tel3cHyd1525a intron, Cm^R^/Tm^R^ | This work |
| pHK-TT1A-Ldh309s | Derived from pHK-TT1A, *E. coli-C. thermocellum* shuttle vector, *groEL* promoter, Tel3cLdh309s intron, Cm^R^/Tm^R^ | This work |
| pHK-TT1A-Ldh508s | Derived from pHK-TT1A, *E. coli-C. thermocellum* shuttle vector, *groEL* promoter, Tel3cLdh508s intron, Cm^R^/Tm^R^ | This work |
| pHK-TT1A-Pta318a | Derived from pHK-TT1A, *E. coli-C. thermocellum* shuttle vector, *groEL* promoter, Tel3cPta318a intron, Cm^R^/Tm^R^ | This work |
| pHK-TT1A-PyrF281s | Derived from pHK, *E. coli-C. thermocellum* shuttle vector, *groEL* promoter, Tel3cPyrF281s intron, Cm^R^/Tm^R^ | This work |
| pIKM1 | Derived from pIMP1, Mls^R^, Amp^R^, Kan^R^, *E. coli-C. thermocellum* shuttle vector | [4] |
| pIMK1PgroEL | Derived from pIKM1, *groEL* promoter, Mls^R^, Amp^R^, Kan^R^, *E. coli-C. thermocellum* shuttle vector | This work |
| pIMK1-TT1A | Derived from pIMK1PgroEL, *E. coli-C. thermocellum* shuttle vector, *groEL* promoter, Tel3c/TeI4c RT targetron cassette, Mls^R^, Amp^R^, Kan^R^ | This work |
| pJIR750ai_GroEL_promoterCelS  pNW33N | *E. coli- Clostridium* shuttle vector, *groEL* promoter, Amp^R^, Cm^R^/Tm^R^  *E. coli-C. thermocellum* shuttle vector, Cm^R^ | [5]  BGSC |
|  |  |  |

1. San Filippo J, Lambowitz AM (2002) Characterization of the C-terminal DNA-binding/DNA endonuclease region of a group II intron-encoded protein. J Mol Biol 324: 933–951. doi:10.1016/S0022-2836(02)01147-6.

2. Mohr G, Ghanem E, Lambowitz AM (2010) Mechanisms used for genomic proliferation by thermophilic group II introns. PLoS Biol 8: e1000391. doi:10.1371/journal.pbio.1000391.

3. Guo H, Karberg M, Long M, Jones JP III, Sullenger B, et al. (2000) Group II introns designed to insert into therapeutically relevant DNA target sites in human cells. Science 289: 452–457. doi:10.1126/science.289.5478.452.

4. Mai V, Lorenz W, Wiegel J (1997) Transformation of *Thermoanaerobacterium* sp. strain JW/SL-YS485 with plasmid pIKM1 conferring kanamycin resistance. FEMS Microbiol Rev 148: 163–167. doi:10.1111/j.1574-6968.1997.tb10283.x.

5. Bannam TL, Rood JI (1993) *Clostridium perfringens*-*Escherichia coli* shuttle vectors that carry single antibiotic resistance determinants. Plasmid 29: 233–235. doi:10.1006/plas.1993.1025.
