# Supplementary material for: A Targetron System for Gene Targeting in Thermophiles and Its Application in Clostridium thermocellum
Source: PLoS One. 2013 Jul 9;8(7):e69032. doi: 10.1371/journal.pone.0069032 (PMC3706431; doi:10.1371/journal.pone.0069032)
Supplement: Table S3 — DNA oligonucleotides used in this study. (DOCX) [file pone.0069032.s005.docx]

**Table S3.** DNA oligonucleotides used in this study.

**TeI3c primers**

Probe172-F 5’ GCAGGACATTCAACATCATC

Probe172-R 5’ GATACTCATCTACAGCCTCTC

Te420f 5’ AACGCGGTAAGCCCGTA

Te680rc 5’ GTTGGTGACCGCACCAGT

TeI3c-3Pst 5’ aaactgcagCGTCATTGTCGGGTAGCAGG

TeI3cEBS1aXXXX 5' CCCCGTaCgCTGANNNNNNAGCAGcGtATCCAATCC

TeI3cEBS2XXXX 5' CGCTAGAAGCCTCGTTANNNNNAGCAGGCCAAAGATGCTG

TeI3cIBS12XXXX 5' aaaactagtAANNNNNNNNNNNNNGTGCGACGCGAAAGCTAG

TeI3cUNI 5' TAACGAGGCTTCTAGCG

**Primers used to change EBS3 and IBS3 in TeI3c**

TeI3cEBS3mutA 5’ TCAGcGtACGGGGACAGCAACTCCGCCGGGGTAaAGTCACCACCTAAC

TeI3cEBS3mutC 5’ TCAGcGtACGGGGACAGCAACTCCGCCGGGGTAcAGTCACCACCTAAC

TeI3cEBS3mutG 5’ TCAGcGtACGGGGACAGCAACTCCGCCGGGGTAgAGTCACCACCTAAC

TeI3cIBS3T-3Pst 5’ aaactgcagCGTCATaGTCGGGTAGCAGG

TeI3cIBS3C-3Pst 5’ aaactgcagCGTCATgGTCGGGTAGCAGG

TeI3cIBS3G-3Pst 5’ aaactgcagCGTCATcGTCGGGTAGCAGG

**Oligonucleotides used to change IBS3 in recipient plasmid target sites**

WT3ctop 5’ gCTGTAGAACCTCTTGAATGGAGACAAGGCAAATGACGGTGGACCAg

WT3cbot 5’ aattcTGGTCCACCGTCATTTGCCTTGTCTCCATTCAAGAGGTTCTACAGctgca

WT3cIBS3Ttop 5’ gCTGTAGAACCTCTTGAATGGAGACAAGGCAtATGACGGTGGACCAg

WT3cIBS3Tbot 5’ aattcTGGTCCACCGTCATaTGCCTTGTCTCCATTCAAGAGGTTCTACAGctgca

WT3cIBS3Ctop 5’ gCTGTAGAACCTCTTGAATGGAGACAAGGCAcATGACGGTGGACCAg

WT3cIBS3Cbot 5’ aattcTGGTCCACCGTCATgTGCCTTGTCTCCATTCAAGAGGTTCTACAGctgca

WT3cIBS3Gtop 5’ gCTGTAGAACCTCTTGAATGGAGACAAGGCAgATGACGGTGGACCAg

WT3cIBS3Gbot 5’ aattcTGGTCCACCGTCATcTGCCTTGTCTCCATTCAAGAGGTTCTACAGctgca

***lacZ* primers**

LacZ30s 5' TCGTTTTACAACGTCGTGA

LacZ1850s 5' TCCAGCGCTGACGGAAGCAA

LacZ1850as 5' TTGCTTCCGTCAGCGCTGGA

LacZ3060as 5' GACACCAGACCAACTGGTAA

**Primers used to target TeI3c to *E. coli lacZ***

LacZ60aIBS12 5’ aaaactagtAAgttgggtaacgccGTGCGACGCGAAAGCTAG

LacZ60aEBS2 5’ CGCTAGAAGCCTCGTTAccaacAGCAGGCCAAAGATGCTG

LacZ60aEBS1a 5’ CCCCGTaCgCTGAaacgccAGCAGcGtATCCAATCC

LacZ369aIBS12 5’ aaaactagtAAatgtgagcgagtaGTGCGACGCGAAAGCTAG

LacZ369aEBS2 5’ CGCTAGAAGCCTCGTTAcacatAGCAGGCCAAAGATGCTG

LacZ369aEBS1a 5’ CCCCGTaCgCTGAcgagtaAGCAGcGtATCCAATCC

LacZ2586aIBS12 5’ aaaactagtAAtcgccatttgaccGTGCGACGCGAAAGCTAG

LacZ2586aEBS2 5’ CGCTAGAAGCCTCGTTAggcgaAGCAGGCCAAAGATGCTG

LacZ2586aEBS1a 5’ CCCCGTaCgCTGAttgaccAGCAGcGtATCCAATCC

**Oligonucleotides used to construct recipient plasmids with *lacZ* target sites**

LacZ60aTOP 5' gTGCTGCAAGGCGATTAAGTTGGGTAACGCCAGGGTTTTCCCAGTCg

LacZ60aBOT 5' aattcGACTGGGAAAACCCTGGCGTTACCCAACTTAATCGCCTTGCAGCActgca

LacZ369aTOP 5' gGCTTTCATCAACATTAAATGTGAGCGAGTAACAACCCGTCGGATTg

LacZ369aBOT 5' aattcAATCCGACGGGTTGTTACTCGCTCACATTTAATGTTGATGAAAGCctgca

LacZ2586aTOP 5' gTTCAACATCAACGGTAATCGCCATTTGACCACTACCATCAATCCGg

LacZ2586aBOT 5' aattcCGGATTGATGGTAGTGGTCAAATGGCGATTACCGTTGATGTTGAActgca

**Primers used for PCR of the *C. thermocellum* *groEL* promoter**

Ct_PgroEL5 5’ ggggatccAGTATTTACTTACAATCGGCG

Ct_PgroEL3 5’ gggaattcctcgagaaaactagtCAACCTGCTTTGGCTTATGTATT

**PCR primers used to construct *E. coli/C. thermocellum* shuttle plasmid pHK**

Phk-F 5’ ccggaattctctagaaagcttggtacccatatggctagcACATACGAGCCGGAAGCATA

Phk-R 5’ cgggaattcctcgagggatccccgggcgccgttctgcagCGAAATAGACAGATCGCTGA

***cipA* primers**

CipA1827sIBS12 5' aaaactagtAAaccgggagaattgGTGCGACGCGAAAGCTAG

CipA1827sEBS2 5' CGCTAGAAGCCTCGTTAccggtAGCAGGCCAAAGATGCTG

CipA1827sEBS1a 5' CCCCGTaCgCTGAgaattgAGCAGcGtATCCAATCC

CipA1827s-F 5’ GCAAAAATAAGAGCAACTG

CipA1827s-R 5’ TCATCTGTCGGTGTTGTTAC

***hfat* primers**

Hfat165sIBS1/2 5' aaaactagtAAtgtcccggcagacGTGCGACGCGAAAGCTAG

Hfat165sEBS1/delta 5' CCCCGTaCgCTGAgcagacAGCAGcGtATCCAATCC

Hfat165sEBS2 5' CGCTAGAAGCCTCGTTAggacaAGCAGGCCAAAGATGCTG

Hfat165s-F 5’ AACGGCATTATGGCTCTT

Hfat165s-R 5’ GCAGACTGACAGGAATAGA

***hyd* primers**

Hyd1525aIBS1/2 5' aaaactagtAAcctttcatcaggaGTGCGACGCGAAAGCTAG

Hyd1525aEBS1/delta 5' CCCCGTaCgCTGAtcaggaAGCAGcGtATCCAATCC

Hyd1525aEBS2 5' CGCTAGAAGCCTCGTTAaaaggAGCAGGCCAAAGATGCTG

Hyd1525a-F 5’ CACCAACACCCAGTTCCTTTAC

Hyd1525a-R 5’ TTACCACCTTTCCTCTCTCACG

***ldh* primers**

Ldh309sIBS1/2 5' aaaactagtAAcacggaagtattcGTGCGACGCGAAAGCTAG

Ldh309sEBS1/delta 5' CCCCGTaCgCTGAgtattcAGCAGcGtATCCAATCC

Ldh309sEBS2 5' CGCTAGAAGCCTCGTTAccgtgAGCAGGCCAAAGATGCTG

Ldh508sIBS1/2 5' aaaactagtAAgtggatgcacgaaGTGCGACGCGAAAGCTAG

Ldh508sEBS1/delta 5' CCCCGTaCgCTGAcacgaaAGCAGcGtATCCAATCC

Ldh508sEBS2 5' CGCTAGAAGCCTCGTTAtccacAGCAGGCCAAAGATGCTG

Ldh309s-F 5’ ACAATGGCTTCAACGATTC

Ldh309s-R 5’ GATGCTCAGCGGACTTAT

***pta* primers**

Pta318aIBS1/2 5' aaaactagtAAgctcatacagtgtGTGCGACGCGAAAGCTAG

Pta318aEBS1/delta 5' CCCCGTaCgCTGAcagtgtAGCAGcGtATCCAATCC

Pta318aEBS2 5' CGCTAGAAGCCTCGTTAtgagcAGCAGGCCAAAGATGCTG

Pta318a-F 5’ TTGGAGTTACTTGCCGTTAT

Pta318a-R 5’ CCATACCATCCGCTTCAC

***pyrF* primers**

PyrF281sIBS1/2 5' aaaactagtAActtgcaaatatgcGTGCGACGCGAAAGCTAG

PyrF281sEBS1/delta 5' CCCCGTaCgCTGAatatgcAGCAGcGtATCCAATCC

PyrF281sEBS2 5' CGCTAGAAGCCTCGTTAgcaagAGCAGGCCAAAGATGCTG

PyrF281s-F 5’ GTGTTATGTAAGGAGAATGA

PyrF281s-R 5’ TTACTTCCTGTCTCGCAACG

Lowercase letters indicate mutations, and N indicates variable bases for DNA targeting. Restriction sites introduced by the oligonucleotides are underlined.
